# Monitoring the Dynamics of Alzheimer's Disease Biomarkers and the *APOE*–Tau Axis via Human Cerebral Organoids with Immuno‐SERS

**DOI:** 10.1002/advs.202505660

**Published:** 2025-05-30

**Authors:** Yongjae Jo, Youngjun Kim, Rian Kang, Seho Lee, Dang Du Nguyen, Soomin Park, Dongjoon Lee, Jong Won Han, Inhee Mook‐Jung, Luke P. Lee, Jong‐Chan Park, Inki Kim

**Affiliations:** ^1^ Department of Biophysics Institute of Quantum Biophysics Sungkyunkwan University Suwon 16419 Republic of Korea; ^2^ Department of Metabiohealth Sungkyunkwan University Suwon 16419 Republic of Korea; ^3^ Department of Biopharmaceutical Convergence Sungkyunkwan University Suwon 16419 Republic of Korea; ^4^ Department of Intelligent Precision Healthcare Convergence Sungkyunkwan University Suwon 16419 Republic of Korea; ^5^ Department of Biochemistry and Biomedical Sciences College of Medicine Seoul National University Seoul 03080 Republic of Korea; ^6^ Convergence Dementia Research Center College of Medicine Seoul National University Seoul 03080 Republic of Korea; ^7^ Department of Medicine Brigham and Women's Hospital Harvard Medical School Boston MA 02115 USA; ^8^ Department of Bioengineering Department of Electrical Engineering and Computer Science University of California Berkeley CA 94720 United States

**Keywords:** APOE, human cerebral organoids (hCOs), surface‐enhanced Raman spectroscopy (SERS), tau

## Abstract

Noninvasive monitoring of Alzheimer's disease (AD) biomarkers is essential for early diagnosis and treatment efficacy. However, noninvasive monitoring of tau protein secretion, a key biomarker of AD, across developmental stages, age‐related variations, and the interaction between apolipoprotein E (*APOE*) and the tau protein axis is not yet accomplished. Here, the label‐free and non‐invasive detection of multiple tau variants dynamics across developmental stages, age‐related variants, and various *APOE* isogenic genotyes is presented to investigate the *APOE*–tau axis using human cerebral organoids (hCOs) combined with surface‐enhanced Raman spectroscopy (SERS). Principal component analysis (PCA) of SERS signals successfully identifies four developmental stages of hCOs: embryonic body, neuronal differentiation, maturation, and maintenance phases. Temporal dynamics of age‐related tau protein secretion are observed, reflecting characteristics associated with AD, which are diminished by astrocyte expression. PCA‐based dimensionality reduction of SERS signals further reveals distinct clustering for different *APOE* isogenic genotypes, with tau protein secretion increasing from *APOE2/E2* to *APOE4/E4*, providing direct insight into the *APOE*–tau axis in AD. This study introduces a novel method for the non‐invasive clinical assessments of disease conditions, dynamics, and the relationship between *APOE* and tau in AD.

## Graphical abstract

Noninvasive and sensitive detection of tau protein dynamics across developmental stages and *APOE* genotypes remain a challenge in Alzheimer's disease (AD) research. This study reveals the dynamics of tau secretion in label‐free manner using surface‐enhanced Raman spectroscopy (SERS) and human cerebral organoids (hCOs). This study provides novel insights into the *APOE*–tau axis in AD.

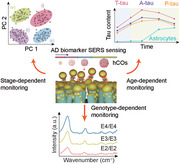

## 1 Introduction

Alzheimer's disease (AD), one of the most prevalent neurodegenerative diseases (NDs) and a leading cause of dementia, is primarily featured by the accumulation of phosphorylated tau (p‐tau) proteins and amyloid beta (Aβ) plaques.^[^ ^1^ ^]^ Although tau protein is a microtubule‐associated protein that stabilizes neuronal structures under normal physiological conditions, its hyperphosphorylated and neurofibrillary tangles (NFT) forms are characteristic features of patients with AD.^[^ ^2^ ^]^ In addition, aggregated tau (a‐tau), which is closely associated with NFT, causes neuronal degradation and synaptic dysfunction, leading to cognitive decline.^[^ ^3^, ^4^, ^5^, ^6^ ^]^ Therefore, NFT is considered as a major hallmark of AD and is targeted for the early diagnosis and therapeutic perspective of AD.^[^ ^7^ ^]^

Apolipoprotein E (*APOE*) gene alleles are the main genetic factors relevant to AD with three isoforms in humans: *APOE2, APOE3, and APOE4*. Among these, *APOE4* is considered the most potent risk factor for sporadic AD, accounting for 87% of AD cases.^[^ ^8^ ^]^ *APOE4* carriers show synaptic dysfunction and neuronal degradation accompanied by increased Aβ and tau aggregation.^[^ ^9^ ^]^ Individuals carrying one *APOE4* allele have double the risk of AD, while those carrying two *APOE4* alleles have triple the risk, compared with individuals carrying two *APOE3* alleles.^[^ ^10^ ^]^ Therefore, uncovering *APOE*‐dependent disease phenotypes is highly needed to understand the pathophysiology of AD deeply.

Owing to limited access to patient brain tissues and the diverse phenotypes of AD, human induced pluripotent stem cells (iPSCs) are considered a valuable platform for designing in vitro disease models of NDs.^[^ ^11^ ^]^ In addition, CRISPR‐Cas9‐based gene editing in iPSC lines enables in vitro investigation of *APOE*‐dependent phenotypes.^[^ ^12^ ^]^ Since it is challenging to reveal direct evidence of the effect of *APOE* genotypes on AD pathology in both human and animal models due to environmental factors,^[^ ^13^ ^]^ human iPSC‐derived human cerebral organoids (hCOs) with the CRISPR‐Cas9 gene‐editing system can offer a promising approach to address these issues.^[^ ^14^ ^]^ Unlike spheroids, cerebral organoids undergo differentiation during the self‐organization of 3D structures in the presence of an extracellular matrix (ECM) and growth factors that recapitulate developmental processes.^[^ ^15^, ^16^, ^17^, ^18^ ^]^ Therefore, this model exhibits key features, such as physiologically relevant 3D structures, cellular diversity, and regional specification of human brain tissue, which align with the widely accepted definitions of brain organoids.^[^ ^12^, ^14^ ^]^

Notably, although previous reports have demonstrated a correlation between *APOE* and tau aggregation in AD, most findings have not been obtained by studies involving the single modulation of *APOE* genotypes.^[^ ^19^, ^20^, ^21^ ^]^ In other words, the effects of independent *APOE* modulation remain unclear. Aging is an important variable to consider. Clinically, cognitive function gradually declines while the risk of NDs increases with age.^[^ ^22^, ^23^, ^24^ ^]^ Moreover, genetic risk factors such as the *APOE* gene are more significant in older individuals than in younger ones.^[^ ^24^, ^25^, ^26^ ^]^ These clinical observations highlight the need for non‐invasive, label‐free, and long‐term monitoring of biomarkers to elucidate the effects of aging on NDs.

*APOE*‐dependent ND biomarker secretion can be analyzed using conventional biomolecule detection techniques such as single molecule array (SIMOA), mesoscale discovery (MSD), liquid chromatography–mass spectroscopy (LC‐MS/MS), and enzyme‐linked immunosorbent assay (ELISA).^[^ ^27^, ^28^, ^29^, ^30^, ^31^ ^]^ Although these techniques provide high sensitivity, they are constrained by complexity, costly inefficiency, and time‐consuming procedures and do not provide detailed information on protein variants or structures. To address these issues, we introduced a highly sensitive surface‐enhanced Raman spectroscopy (SERS) based immunoassay. SERS enables sensitive biomolecule detection by amplifying Raman signals at plasmonic “hot spots”, where the electric field is locally enhanced.^[^ ^32^, ^33^ ^]^ It provides high‐dimensional molecular fingerprint data related to both the protein structure and quantity.^[^ ^34^, ^35^ ^]^ Here, we report a non‐invasive and label‐free SERS‐based platform for detecting tau protein variants, which are key AD biomarkers, secreted from hCOs, thereby demonstrating the feasibility of the proposed in vitro approach for studying AD. We prepared isogenic hCOs, spanning *APOE2/E2* to *APOE4/E4*, using CRISPR‐Cas9 gene editing. We then performed temporal monitoring of AD biomarkers dynamics to investigate age‐ and genotype‐dependent secretion using gold‐coated zinc oxide (ZnO) nanorod substrates conjugated with a half‐antibody for SERS. By incorporating dimensionality reduction techniques, quantitative and structural information on biomarkers can be extracted from the SERS spectrum, offering meaningful insights into AD progression and neuropathogenic dynamics.^[^ ^36^ ^]^

**Figure 1:**
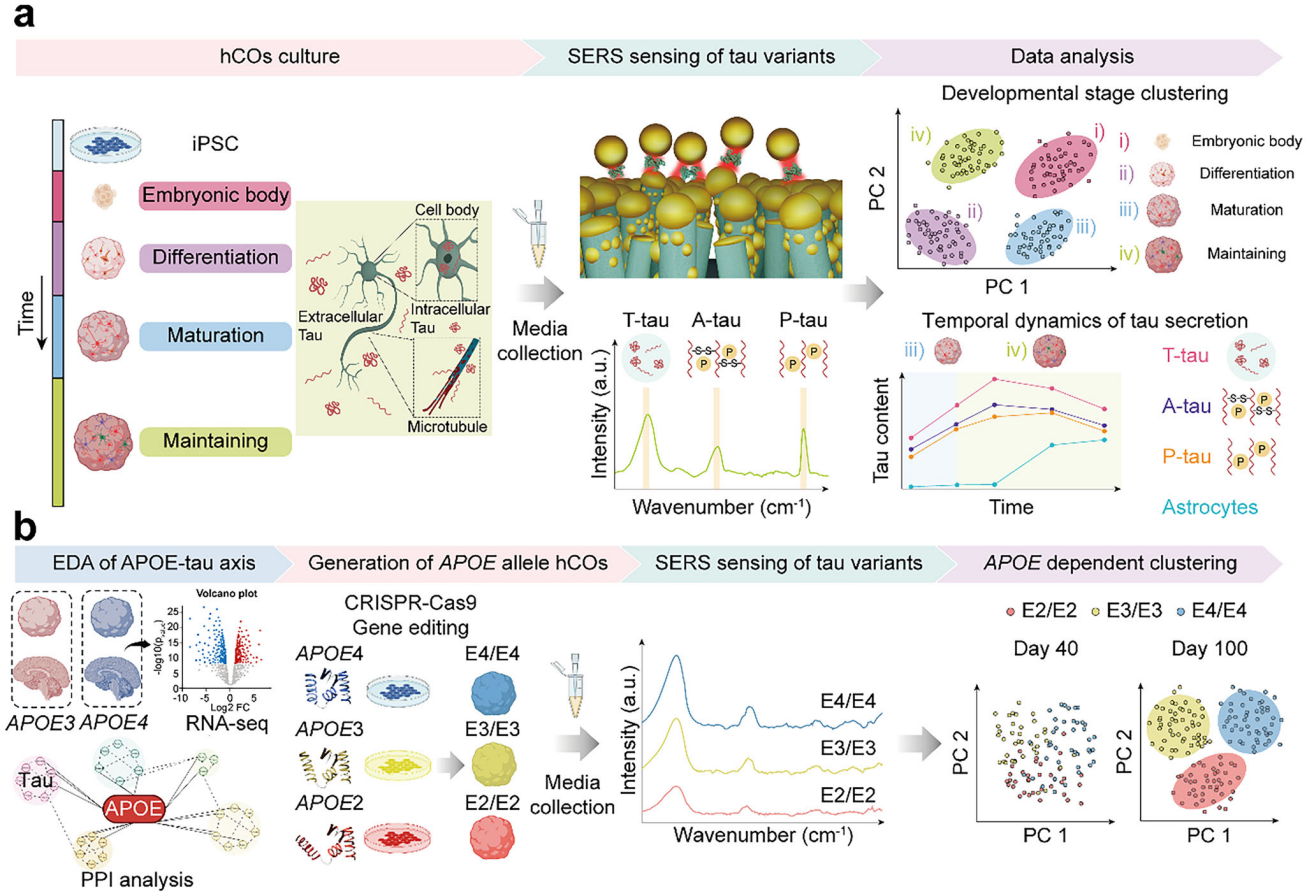
Scheme of SERS sensing and PCA analysis for tau variants secreted from human cerebral organoids (hCOs). a) Schematic of SERS sensing for tau protein variants with SERS based PCA clustering according to hCOs developmental stages and temporal monitoring. b) Schematic of SERS sensing of tau variants secreted from *APOE*‐dependent generated hCOs and PCA clustering. hCOs, human cerebral organoids; iPSC, induced pluripotent stem cells; SERS, surface enhanced Raman spectroscopy; T‐tau, total‐tau; A‐tau, aggregated tau; P‐tau, phosphorylated‐tau; PC, principal component; EDA, exploratory data analysis; *APOE*, apolipoprotein E; RNA‐seq, RNA‐sequencing; PPI, protein‐protein interaction.

As a proof of principle, we first validated the temporal dynamics of tau secretion using our SERS‐based platform in *APOE3/E4* hCOs (**Figure** **1**a). By applying principal component analysis (PCA)‐based dimensional reduction to the SERS spectra, we successfully distinguished the four developmental stages of hCOs. In addition, temporal quantification of the biomarker revealed an age‐dependent increase in tau protein secretion, reflecting characteristics related to neurodegeneration, which was attenuated by the gradual expression of astrocytes through endocytosis.^[^ ^37^, ^38^ ^]^ Finally, we observed distinct clusters of SERS spectra for each *APOE* isogenic hCOs line, with tau protein secretion increasing from *APOE2/E2* to *APOE4/E4* (Figure 1b). Notably, these genotype‐dependent clusters and the corresponding increase in biomarker secretion were more pronounced on day 100 than on day 40, underscoring the age‐related features of NDs. We believe this label‐free, non‐invasive approach for biomarker detection in hCOs using SERS can potentially enable robust clinical assessments of disease progression and provide direct insights into the *APOE*–tau axis in AD.

## 2 Results and Discussion

### 2.1 Generation of Organoids and Optimization of SERS Substrates

To evaluate the non‐invasive detection of AD biomarker dynamics, we optimized both organoid generation and the fabrication of SERS substrates for label‐free and non‐invasive measurements. The hCOs were cultured from human iPSCs, starting at the embryonic body (EB) stage and continuing for ≈120 days (see Experimental Section and Figure S1, Supporting Information). To determine whether the organoids possessed cortical cell populations, such as neurons, astrocytes, and neural progenitor cells (NPCs), the cultured organoids were validated by immunostaining using cell type‐specific antibodies (Figure S1, Supporting Information). Meanwhile, we fabricated gold‐coated ZnO nanorods functionalized with half‐antibodies to detect tau proteins (Figure S2, Supporting Information). The substrate for SERS was optimized by varying the seeding method, pre‐heating temperature, and reaction time, followed by 100 nm gold deposition. Through SERS measurements of rhodamine 6G (R6G), the optimal fabrication conditions for the best SERS performance were identified as 300 °C pre‐heated spin coating seeding and 3 hours reaction time (Figures S3 and S4, Supporting Information). Similarly, we also determined the optimal concentrations for the tau primary antibody (0.16 mg mL^−1^) and tris(2‐carboxyethyl)phosphine (TCEP) (0.02 mm), which cleaves antibody to generate thiol groups for conjugating half‐antibodies onto the SERS substrates (Figure S5, Supporting Information).

To enhance the sensitivity, we adopted nanotags made of gold nanoparticles (GNPs) conjugated half‐antibodies, which create additional localized electric fields between the substrates and GNPs. This resulted in a 1.65‐fold enhancement in SERS signals compared with conditions without nanotags (Figures S2 and S6, Supporting Information).^[^ ^39^ ^]^ We performed finite‐difference time‐domain (FDTD) electromagnetic simulations to examine the effects of nanotags numerically. The gold‐coated ZnO nanorods SERS substrate was statistically modeled by randomly sampling the structural parameters obtained from scanning electron microscopy (SEM) images (see Experimental Section and Figures S4 and S7, Supporting Information). The simulation results showed that the GNPs nanotags generated additional hot spots near the nanorods, enhancing the local electric field by approximately 83 times compared with the cases without nanotags (Figure S8, Supporting Information).^[^ ^40^, ^41^ ^]^

To quantitatively monitor distinct tau protein variants, we selected three Raman bands—300, 500, and 1050 cm⁻¹—corresponding to total‐ (t‐), aggregated‐ (a‐), and hyperphosphorylated (p‐) tau proteins, respectively. The 300 cm⁻¹ peak, attributed to the gold–thiol bond,^[^ ^40^, ^41^, ^42^ ^]^ was chosen for t‐tau detection owing to its strong and stable signal derived from gold–thiol bonding at tau antibody functionalized GNP nanotags that specifically bind to tau proteins. This Raman fingerprint is robust against interference from biological molecules, ensuring stable and reliable measurements.^[^ ^42^ ^]^ Calibration experiments using both monomeric and fibrillar tau proteins confirmed a linear relationship between the concentration and signal intensity at the 300 cm^−1^ band. Further quantitative analysis with fibrillar tau at this band demonstrated an exceptionally low limit of detection (LOD) (7.4 fM) and high spatial mapping uniformity (Figure S9, Supporting Information). This sensitivity is sufficient to detect tau proteins in human blood samples, including both normal (≈200 fM) and AD patients, who exhibit 2–2.5 times higher tau levels.^[^ ^43^ ^]^

To assess other tau variants, we calibrated additional Raman bands at 500 and 1050 cm⁻¹. The 500 cm⁻¹ peak, corresponding to disulfide bonding,^[^ ^44^ ^]^ was selected for a‐tau quantification, as intermolecular disulfide bonds play an important role in tau aggregation.^[^ ^45^ ^]^ SERS signals at this band exhibited a clear linear correlation with the protein concentration, which was stronger for fibrillar tau (R^2^ = 0.98) than that for monomeric tau (R^2^ = 0.65) (Figures S9 and S10, Supporting Information). Although tau aggregation can occur independent of phosphorylation,^[^ ^45^, ^46^, ^47^, ^48^, ^49^, ^50^, ^51^ ^]^ this difference may be attributed to the fact that phosphorylation further promotes aggregation process.^[^ ^52^, ^53^, ^54^ ^]^ Consequently, the hyperphosphorylated fibrillar tau exhibited more distinct aggregation features in the calibration results. For p‐tau detection, we utilized the 1050 cm⁻¹ phosphate stretching band,^[^ ^55^ ^]^ a hallmark of hyperphosphorylated tau that is closely associated with the formation of NFTs and AD. Notably, this band showed a distinct concentration‐dependent increase in the signal for fibrillar tau (R^2^ = 0.98), whereas monomeric tau, which lacks hyperphosphorylation, exhibited a negligible signal and weak correlation (R^2^ = 0.29) (Figures S9 and S10, Supporting Information). These results evidently confirmed the molecular specificity of our band selection strategy and demonstrated its utility in the sensitive and multiplexed detection of tau variants relevant to AD pathology.

Although tau‐specific antibodies were employed in this study, SERS measurements in complex biological samples can be disrupted by external chemical species, such as non‐targeted proteins, a phenomenon known as the matrix effect.^[^ ^56^ ^]^ To evaluate this potential interference, we performed a recovery test, which is a widely accepted standard for validating SERS reliability in the presence of interfering chemical species.^[^ ^57^, ^58^ ^]^ Standard fibrillar tau solutions (100 pM, 1 pM, and 10 fM) were spiked into organoid culture media, which contains a variety of extracellular proteins and growth factors. Following SERS measurements, the recovered concentrations were estimated using previously established calibration curves (Figures S9 and S11, Supporting Information). The resulting recovery rates, calculated as (measured/target concentration) × 100%, ranged from 87% to 109% with relative standard deviations <4.5% (Table S1, Supporting Information). These findings demonstrate that our SERS‐based platform enables the accurate quantification of tau proteins even in complex media by effectively mitigating matrix effects through antibody‐based specificity.

### 2.2 Detection of Tau Proteins Secreted from Various Developmental Stages of hCOs

Defining the various differentiation states of hCOs is critical for the quality control of hCOs and understanding their developmental progress. Monitoring developmental dynamics through biomarker detection enhances our understanding of hCOs maturation and provides essential insights into developmental biology and NDs. Although conventional sensing techniques, including SIMOA, MSD, and ELISA, provide sufficient sensitivity, they necessitate labelling and sample destruction or involve complexity, high costs, and time‐consuming processes.^[^ ^14^, ^59^ ^]^ These limitations hinder sustained long‐term temporal monitoring, which is vital to uncover the effects of aging and development in ND research.

**Figure 2:**
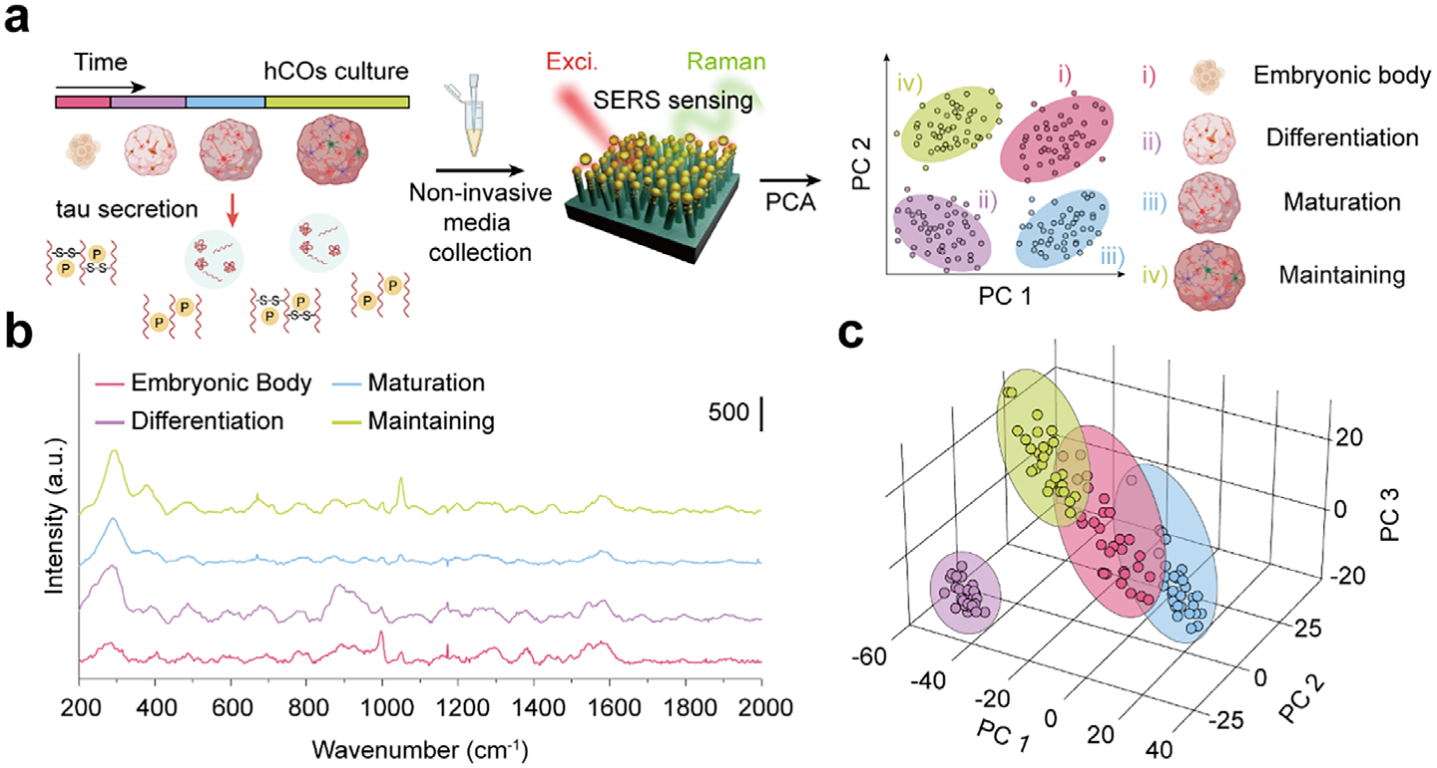
SERS spectra‐based PCA analysis for various hCOs differentiation stages. a) Schematic of PCA analysis based on SERS signal for tau protein secreted from various hCOs differentiation stages. b) Mean SERS spectra of cultured media collected from each hCOs differentiation stage. Each spectrum represents the average of multiple measurements for each differentiation stage. c) 3D PCA scatter plot based on SERS spectra of each hCOs differentiation stage.

To address these limitations, we utilized SERS‐based immunoassay. We obtained Raman spectra from non‐invasively collected culture media of developing hCOs, which are classified into four distinct stages: EBs, neuronal differentiation, neuronal maturation, and maintaining stage.^[^ ^15^ ^]^ We conjugated tau primary antibodies on the SERS substrates to investigate the secretion profiles of tau proteins across various developmental stages of hCOs. This approach enabled us to analyze the secretion of biomarkers at each developmental stage without labelling by examining the Raman profiles of biomarkers. As high‐dimensional Raman spectra sparsely contain useful biomolecular information, PCA‐based dimensional reduction was applied to the spectra for clustering (**Figure** **2**a).

The hCOs culture media were collected at distinct developmental time points for SERS measurements, specifically on days 4, 20, 40, and 100, which correspond to the EBs, neuronal differentiation, neuronal maturation, and maintenance stages, respectively (Figure 2b). By applying PCA to the obtained Raman spectra, we successfully identified distinct clusters, assessed by a silhouette coefficient of 0.41, an indicator of clustering quality,^[^ ^60^, ^61^ ^]^ with each cluster corresponding to a specific developmental stage (Figure 2c). It is important to note that although we selected only three principal components, they accounted for as much as 96.2% of the variance ratio. Notably, the PCA loading values highlight the spectral regions that contribute most to this discrimination, particularly the peaks at 300, 700, and 1050 cm^−1^, which are difficult to distinguish visually in the raw Raman spectra (Figure S12, Supporting Information). The peaks at 300 and 1050 cm⁻¹ correspond to t‐tau and phosphorylation, respectively, whereas the 700 cm⁻¹ peak is recognized as a fingerprint for C─S stretching vibrations and methionine residues in proteins.^[^ ^62^ ^]^ These results highlight a strong correlation between the tau protein secretion profile and developmental stages of hCOs. Notably, the distinct clustering observed in the PCA space suggests the versatility of SERS‐based biomarker analysis, enabling the rapid and efficient discrimination of biological states in hCOs. This label‐free and non‐invasive approach serves as a reliable method for biomarker analysis in tracking the maturation of hCOs, as well as for the precise monitoring of tau protein secretion dynamics.

### 2.3 Temporal Dynamics of Tau Protein Secretion from hCOs

**Figure 3:**
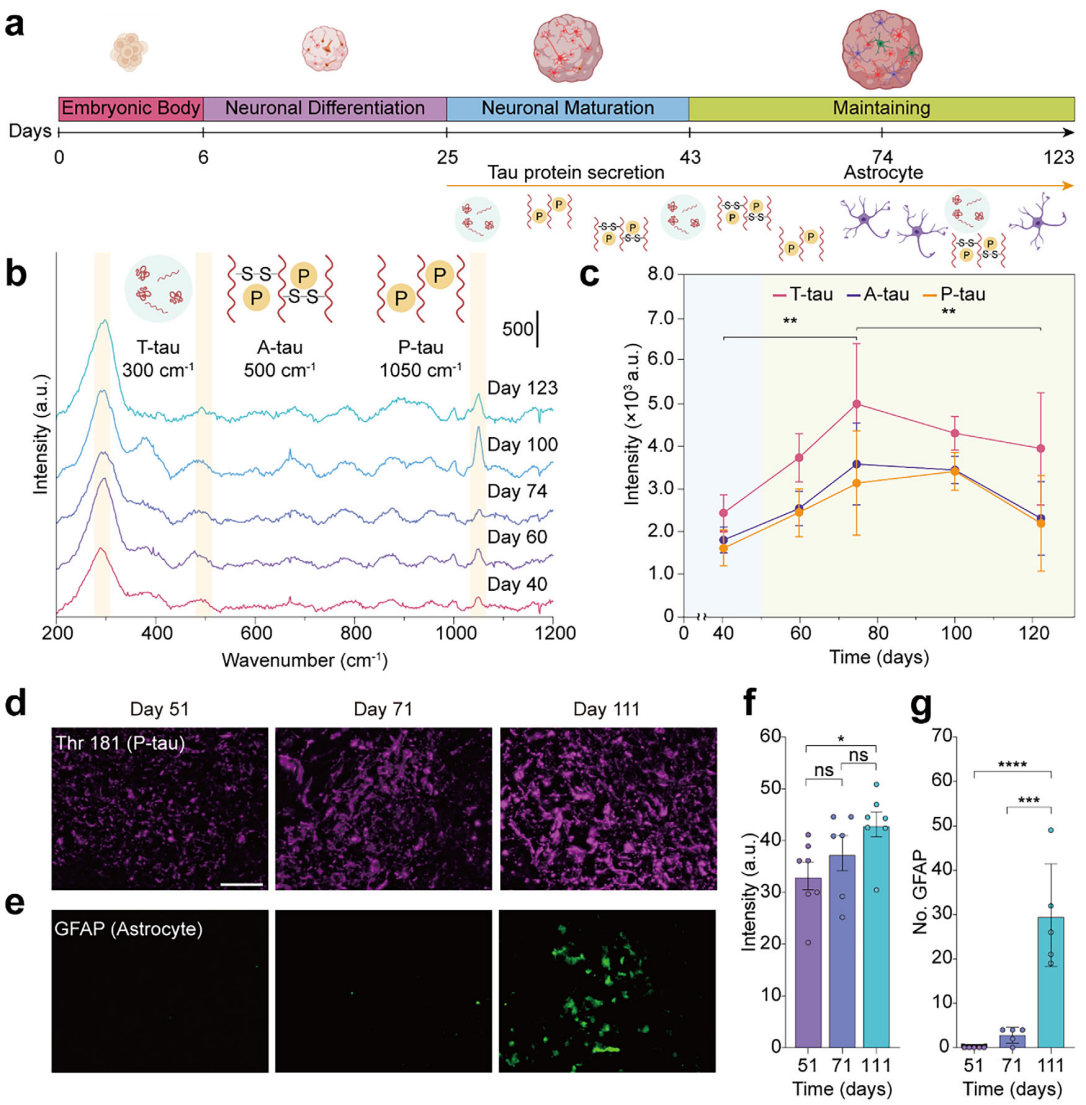
Temporal monitoring of tau protein secreted from hCOs via SERS and IHC validation. a) Schematic of temporal monitoring of tau protein secretion from hCOs at different days. b) SERS spectra of hCOs at days 40, 60, 74, 100, and 123. c) Day‐response curves according to SERS signal of tau protein at 300, 500, and 1050 cm^−1^, indicating t‐tau, a‐tau, and p‐tau, respectively, from the late stage of neuronal maturation stage day 40 (shown in blue) to the maintaining stage day 123 (shown in green). Error bars indicate ± SD. Data are presented as mean values ± SD. d) Immunohistochemistry showing p‐tau (Thr181) in parental hCOs. e) Immunohistochemistry showing astrocyte (GFAP) in parental hCOs. f) Fluorescence quantification of p‐tau on days 51,71, and 111 hCOs. Data are presented as mean ± s.e.m.; *n* = 6,7, and 6 independent slices for days 51, 71, and 111 hCOs lines, respectively. Brown‐Forsythe ANOVA with the correction of Tamhane T2 post‐hoc test for multiple comparisons. g) Quantification of GFAP signals. Data are presented as mean ± s.e.m.; *n* = 5, 5, 5 independent slices for days 51, 71, and 111 hCOs lines, respectively. Welch's ANOVA with the correction of Tukey's post hoc test for multiple comparisons. ****p < 0.0001; ***p < 0.001; **p < 0.01; *p < 0.05; ns, non‐significance. Scale bar: 100 µm (d).

Secretion of tau proteins increases with neuronal maturation, which is highly associated with AD pathogenesis (**Figure** **3**a).^[^ ^63^ ^]^ Thus, quantitative and qualitative long‐term measurements of tau secretion are demanded for clinical studies on ADs. As previously mentioned, SERS provides a fast and non‐invasive approach for the time‐series acquisition of biomarker secretion with high sensitivity (Figure S9, Supporting Information).^[^ ^64^ ^]^ Although the tau protein stabilizes neuronal structures under physiological conditions, it becomes hyperphosphorylated and aggregated, eventually forming NFT structures in AD patients. These aggregated forms lead to neuronal degradation and synaptic dysfunction, which ultimately contribute to cognitive decline.^[^ ^3^, ^4^, ^5^ ^]^ In addition, the t‐tau level is critical in individuals with AD due to neuronal plasticity, blood‐brain barrier dysfunction, and blood‐cerebrospinal fluid barrier dysfunction.^[^ ^65^ ^]^ Consequently, t‐tau proteins, including the p‐ and a‐ forms, are widely regarded as major neuropathological features^[^ ^66^ ^]^ and markers for the early diagnosis of AD.^[^ ^7^ ^]^

In many current approaches, tau pathology in AD has been studied by measuring p‐tau, which is known to induce NFT formation and neurotoxicity.^[^ ^14^ ^]^ However, simultaneous measurement of each tau variant and its ratio is essential for studying AD progression, as NFT maturation in AD follows a well‐defined pattern of phosphorylation.^[^ ^67^ ^]^ The proposed SERS‐based immunoassay can reveal the correlation between secreted tau protein variants, including t‐, a‐, and p‐tau, through individual molecular fingerprints of the SERS spectra. The 300,^[^ ^40^ ^]^ 500,^[^ ^68^ ^]^ and 1050^[^ ^55^ ^]^ cm^−1^ Raman peaks were the markers for quantitative measurements of t‐, a‐, and p‐tau, respectively. The 300 cm^−1^ peak, attributed to the gold**─**thiol bond, is a t‐tau marker because GNP nanotags featuring gold–thiol bonds exclusively bind to tau proteins.^[^ ^40^ ^]^ The disulfide bond peak at 500 cm^−1^ was adopted for a‐tau detection because tau aggregation involves numerous disulfide bonds.^[^ ^68^, ^69^ ^]^ Similarly, the 1050 cm^−1^ was used as a direct marker for p‐tau since it is reported as a feature of p‐tau.^[^ ^55^ ^]^ For temporal monitoring, we selected time points from day 40, just before neuronal maturation completes, to day 123 of the maintenance stage (Figure 3b). Notably, we also sampled the SERS spectra on day 74, when astrocyte differentiation begins.

Quantification of the selected peaks showed that all tau variants had similar profiles over time (Figure 3c). From day 40 to day 74, the amounts of t‐, a‐, and p‐tau increased since neuronal maturation almost finished at approximately day 70.^[^ ^15^ ^]^ Interestingly, however, the levels of all tau protein variants began to slightly decrease after day 74 and eventually exhibits a significant decrease by day 123.^[^ ^15^ ^]^ We reasoned that the significant reduction in tau protein levels is attributed to astrocyte differentiation, given their known functions in the uptake of molecular wastes such as tau proteins.^[^ ^70^ ^]^ To validate this hypothesis, we performed immunohistochemical staining (IHC) of hCOs tissues on days 51, 71, and 111 using Thr 181 (p‐tau marker) and GFAP (astrocyte marker) antibodies (Figure 3d,e). The gradual increase in p‐tau expression over the culture period exhibited a trend similar to that of the time‐dependent observations in the SERS data (Figure 3c,f), supporting the feasibility of our SERS‐based platform for biomarker screening. Moreover, the astrocyte count data revealed that astrocytes began to appear on day 71, followed by a sudden increase on day 111, when a slight decrease in tau protein variants was observed in the SERS data (Figure 3c,g).^[^ ^15^ ^]^ This evidence supports our hypothesis that astrocytes uptake and remove tau proteins, which is consistent with previous results regarding the function of astrocytes in waste clearance.^[^ ^37^, ^71^ ^]^

Our SERS‐based immunoassay provided a comprehensive time‐dependent analysis of tau secretion, highlighting the age‐dependent secretion of AD biomarkers. Molecular fingerprints in the SERS spectra enabled the simultaneous identification of distinct secretion profiles for t‐, a‐, and p‐tau over the culture period, demonstrating the feasibility of our platform for biomarker screening in clinical studies on AD. Furthermore, the effects of other cell types—in this case, astrocytes—was evident in our measurements, emphasizing the versatility of this technique. We expect that these advantages will make the proposed SERS‐based immunoassay a versatile platform for the long‐term monitoring of biomarkers.

### 2.4 Exploring *APOE*‐Tau Axis Through RNA Sequencing and Protein–Protein Interaction (PPI) Analysis

In AD pathology, it is well‐known that the *APOE* genotypes are strongly associated with AD risk. Specifically, *APOE4* is considered the most significant risk factor, accounting for 87% of AD cases.^[^ ^8^ ^]^ Individuals with one *APOE4* allele have twice the risk of AD, while those with two *APOE4* alleles have three times the risk compared with individuals with two *APOE3* alleles.^[^ ^10^ ^]^ Therefore, identifying biomarker expression patterns associated with *APOE* genotypes offers valuable insights into the impact of *APOE* genotypes on AD progression.

**Figure 4:**
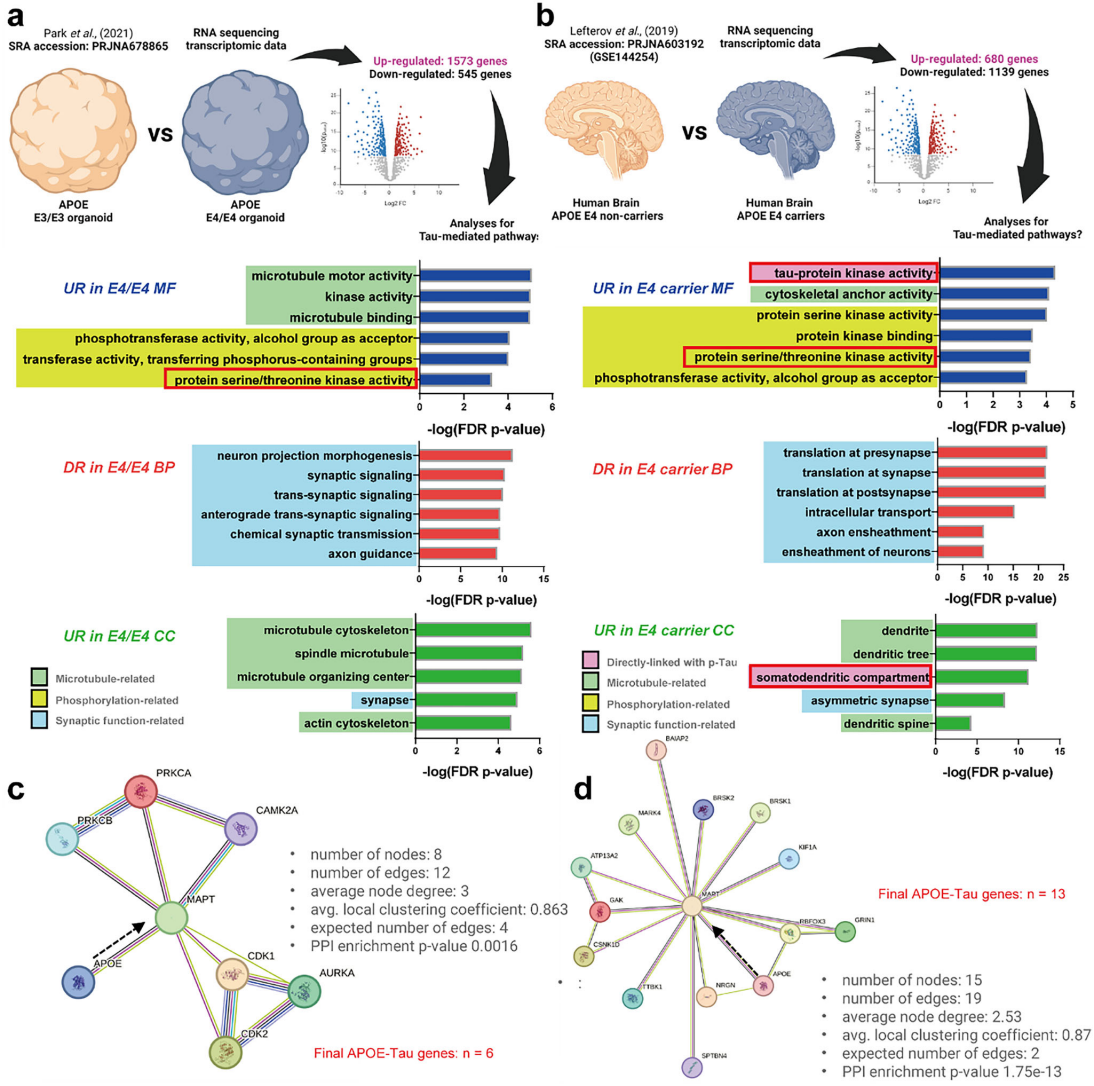
Transcriptomic analyses for brain organoids (*APOE3/E3* versus *E4/E4)* and human brain (*E4* non‐carriers versus *E4* carriers) to reveal the possible *APOE*–tau axis genes. a) RNA sequencing analysis (SRA accession number: PRJNA678865) for human brain organoids (*APOE3/E3* versus *E4/E4)* and ToppGene‐based Gene Ontology (GO) analysis. 1573 up‐regulated differentially expressed genes (DEGs) (log_2_FC > 1) and 545 down‐regulated DEGs (log_2_FC <‐1) were used for GO analysis (FDR‐corrected *p‐*value < 0.05). The term enclosed in the red rectangle was used for protein–protein interaction (PPI) analysis. UR, up‐regulated; DR, down‐regulated; MF, molecular function; BP, biological process; CC, cellular component; FDR, false discovery rate. b) RNA sequencing analysis (SRA accession number: PRJNA603192) for human postmortem brain tissues (*APOE4* non‐carriers versus *E4* carriers) and ToppGene‐based Gene Ontology (GO) analysis. 680 up‐regulated DEGs and 1139 down‐regulated DEGs (adjusted *p*‐value < 0.1) were used for GO analysis (FDR‐corrected *p‐*value < 0.05). The terms closed in red rectangles were used for PPI analysis. UR, up‐regulated; DR, down‐regulated; MF, molecular function; BP, biological process; CC, cellular component; FDR, false discovery rate. c) PPI analysis by STRING database for brain organoids. Selected *APOE*‐tau genes were used for mapping. The result of full PPI mapping is shown in Figure S13 (Supporting Information). d) PPI analysis by STRING database. Selected *APOE*–tau genes were used for mapping. The result of full PPI mapping is shown in Figure S14 (Supporting Information).

We used RNA sequencing data to highlight the direct association between *APOE* and tau formation and to explore the correlation of *APOE* with tau forms using SERS. We investigated the potential genes associated with the APOE–tau axis. For gene ontology (GO) and PPI analyses, two publicly available transcriptomic datasets (Cohort 1: RNA sequencing for brain organoids, *APOE3/E3* versus *APOE4/E4*, SRA number: PRJNA678865; Cohort 2: RNA sequencing for human postmortem brain tissues, *APOE4* non‐carriers versus carriers, each *n* = 20 and *n* = 22, SRA number: PRJNA603192) were used (**Figure** **4**). First, we found that both transcriptomic data from human brain organoids and brain tissues were highly associated with tau‐ and p‐tau‐related terms (microtubule binding, protein serine/threonine kinase activity, somatodendritic compartment, etc.) and synaptic functions (synaptic signaling, anterograde trans‐synaptic signaling, translation of synapse, asymmetric synapse, etc.) (Figure 4a,b). In addition, as both transcriptomic datasets were associated with kinase activity pathways directly related to p‐tau (protein serine/threonine kinase pathways, tau‐protein kinase activity, and somatodendritic compartment), we performed PPI analysis to identify genes directly connected to MAPT, the gene responsible for tau expression (selected mapping results, Figure 4c and d; full mapping results, Figures S13a and S14a, Supporting Information). Although no overlapping genes were identified between the organoids and human brain, we found 6 (Figure 4c; Figure S13b, Supporting Information; red colored genes) and 13 (Figure 4d; Figure S14b, Supporting Information; red colored genes) genes directly associated with the *APOE*–tau axis in the organoids and the human brain, respectively. These transcriptomic data strongly support the need for research on the direct axis of *APOE*–tau. We aimed to demonstrate the direct correlation between *APOE* and tau forms through iPSC‐driven hCOs with CRISPR‐Cas9‐mediated *APOE* gene editing and SERS.

### 2.5 Characterization of Tau Proteins Variants Across Different *APOE* Genotypes in hCOs via SERS

Next, we demonstrated that the detection of altered AD biomarkers was dependent on the *APOE* genotype using SERS on different culture dates. RNA sequencing and PPI analysis provided direct and critical correlations, supporting the necessity of investigating the *APOE*–tau axis. To identify the direct *APOE*–tau axis non‐destructively, we utilized SERS for tau variants detection across various *APOE* allele comparisons of immature and mature states of *APOE* gene–edited hCOs.

**Figure 5:**
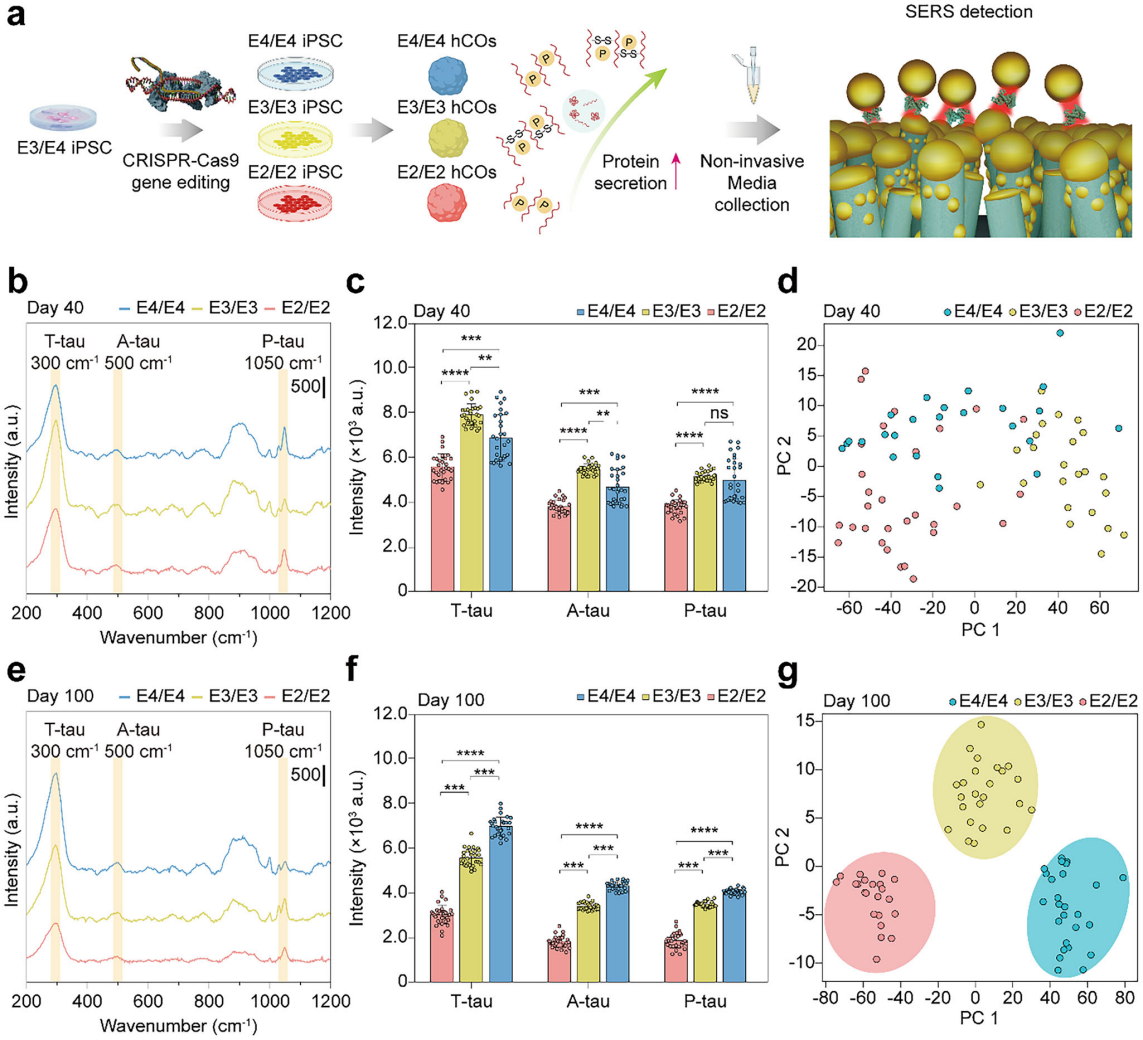
SERS measurements of tau protein secreted from hCOs at different days (days 40, and 100) and *APOE* genotypes (*APOE2*/*E2*, *E3/E3*, and *E4/E4*). a) Schematic of CRISPR‐Cas9 gene edited *APOE* genotype hCOs generation and SERS measurement of tau variants secreted from hCOs with different *APOE* genotypes. b) SERS signal of tau variants acquired from immature (day 40) hCOs with different *APOE* genotypes. c) Quantification of SERS signals at specific peaks obtained from b, representing t‐tau (300 cm^−1^), a‐tau (500 cm^−1^) and p‐tau (1050 cm^−1^). Data are presented as mean values ± SD. d) SERS spectra based PCA clustering according to different *APOE* genotypes in immature state hCOs. e) SERS signal of tau variants acquired from mature (day 100) hCOs with different *APOE* genotypes. f) Quantification of SERS signals at specific peaks obtained from e, representing t‐tau (300 cm^−1^), a‐tau (500 cm^−1^), and p‐tau (1050 cm^−1^). Data are presented as mean values ± SD. g) SERS spectra based PCA clustering according to different *APOE* genotypes in mature state hCOs. Kruskal‐Wallis test and Dunn's test for multiple comparisons was used. ****p < 0.0001; ***p < 0.001; **p < 0.01; ns, non‐significance. *n* = 3, 3, 3 independent hCOs for *APOE2/E2*, *E3/E3*, *E4/E4* hCOs lines, respectively.

To demonstrate the feasibility of the SERS‐based platform, we acquired SERS spectra from the culture media collected from hCOs with different *APOE* genotypes at different time points (**Figure** **5**a). Immature (day 40) and mature (day 100) hCOs were prepared to mimic the aging effect under different genetic conditions to investigate single modulation *APOE* genotype via CRISPR‐Cas9 gene editing techniques. (*APOE2/E2*, *E3/E3*, and *E4/E4*). In the immature state, *APOE3/E3* hCOs secreted the highest amount of tau proteins with statistical significance regardless of the variant types, except for p‐tau, where *APOE4/E4* hCOs showed a slightly lower level than *APOE3/E3* without statistical significance (Figure 5b,c). We conducted PCA to visualize the biomarker secretion patterns utilizing two principal components that explained as much as 95.32% of the variance. The PCA results in the immature state showed no clear genotype‐dependent clusters, as indicated by the low silhouette coefficient of 0.24, which evaluates the clustering quality. Typically, values between 0.5 and 1 indicate well‐defined clusters, whereas values between 0.25 and 0.5 suggest weak clustering (Figure 5d).^[^ ^60^, ^61^ ^]^ This implies that genetic risk is not clearly represented in the immature state.

By contrast, mature hCOs exhibited a consistent and statistically significant monotonic increase in tau protein secretion with increasing genetic risk, regardless of the variant type, from *APOE2/E2* to *APOE4/E4*. Specifically, *APOE4/E4* isogenic hCOs exhibited the highest levels of t‐, a‐, and p‐tau secretion, whereas *APOE2/E2* isogenic hCOs showed the lowest levels of protein secretion (Figure 5e,f). These trends were reflected in the PCA results, which showed clear genotype‐dependent clusters with a high silhouette coefficient of 0.67, and the two principal components explained 96.79% of the variance (Figure 5g). This result highlights the fact that the genetic risk associated with *APOE4/E4* has a significant impact on the mature state hCOs, which is a characteristic feature of NDs. We would like to emphasize that SERS‐based platforms non‐invasively and rapidly identified the genotype‐dependent biomarkers detection at different time points.

We also collected hCOs corresponding to the conditioned media to validate our technique and performed IHC using the MAP2 and Thr181 antibodies, which serve as neuronal and p‐tau markers, respectively (Figure S15, Supporting Information). We then quantified the protein expressions in 3D using the IMARIS software to compare the extra‐ and intracellular p‐tau content with the MAP2 boundary as a reference to define extra‐ and intracellular p‐tau. As a result, the total p‐tau content progressively increased with genetic risk, from *APOE2/E2* to *APOE4/E4*. Notably, the *APOE3/E3* and *APOE4/E4* groups exhibited significantly larger amounts of p‐tau than the *APOE2/E2* group, whereas the difference in intracellular p‐tau levels was not statistically significant (Figure S15, Supporting Information). These findings suggest that *APOE3/E3* and *APOE4/E4* hCOs exhibit increased p‐tau secretion compared with the protective *APOE2/E2* genotype. These tau protein secretion profiles were consistent with the SERS results, providing direct evidence for the *APOE*–tau axis.

## 3 Conclusion 

In this study, we present a highly sensitive, non‐invasive, and label‐free platform based on SERS for the detection of tau protein variants, which are key AD biomarkers, from hCOs. We monitored the temporal dynamics of biomarker secretion from *APOE* isogenic hCOs with genes edited using CRISPR‐Cas9. While conventional biomarker detection methods, such as ELISA and SIMOA, provide sufficient sensitivity, they rely on invasive, labelling‐dependent techniques, and are limited by complexity, high costs, and time‐consuming procedures.^[^ ^72^, ^73^ ^]^ By contrast, the proposed SERS platform preserves the native biological state of biomarkers, enabling the accurate and efficient detection of biomarker secretion dynamics.

As a proof of concept, we investigated the temporal dynamics and *APOE* genotype‐dependent biomarker secretion using the proposed SERS‐based immunoassay. We successfully classified the developmental stages of *APOE3/E4* hCOs by applying PCA to the SERS spectra of the secreted tau proteins. Additionally, we observed age‐related increases in tau protein secretion, which reflect the characteristics of NDs, followed by a decrease in secretion associated with astrocytes differentiation. Finally, we acquired SERS spectra from *APOE* isogenic hCOs in both the immature and mature states, which revealed increased tau protein secretion as the genetic risk increased from *APOE2/E2* to *APOE4/E4*.^[^ ^8^, ^74^, ^75^ ^]^ Notably, distinct clusters corresponding to each *APOE* genotype were identified from the SERS spectra of mature hCOs using PCA, whereas the SERS spectra from immature hCOs lacked clearly defined clusters. These results align with our intuition and those of previous reports, indicating that genetic risk becomes more significant with age. Our findings underscore the application of CRISPR‐Cas9 in human iPSC‐derived organoid models to investigate *APOE* genotype‐specific effects on tau pathology using SERS, suggesting a robust platform for advancing AD research.

Although our study focused on tau protein secretion, the proposed technique is highly versatile and adaptable for the detection of biomarkers associated with a broader range of diseases. A label‐free and temporal analysis of multiple AD biomarkers secreted from hCOs could provide a more comprehensive understanding of AD pathology. These advancements have the potential to support the development of novel diagnostic platforms and enhance drug screening processes for NDs. As one of the ultimate goals of our platform is clinical applications, a comprehensive discussion is required. While our platform demonstrated excellent performance in hCOs‐derived media, translating it into clinical use presents several challenges. First, clinical application requires strict ethical and procedural compliance. Institutional Review Board (IRB) approval is mandatory for acquiring patient‐derived samples and conducting human subject research, requiring rigorous documentation and review processes. Second, our hCOs models provide isogenic backgrounds via CRISPR‐Cas9 editing, enabling controlled investigations of specific genetic factors. However, such control is not feasible in clinical samples, which are affected by individual variability in age, genetic background, and comorbidities. To address this issue, advanced dimensionality reduction techniques and artificial intelligence (AI)‐based approaches have gained attention for their capacity to manage complex biological variability. Such approaches facilitate the identification of subtle patterns within high‐dimensional datasets, which are often difficult to capture using conventional methods. Third, clinical biofluids such as cerebrospinal fluid (CSF) and blood are compositionally complex, containing diverse proteins, metabolites, and extracellular vesicles that can interfere with SERS specificity. Although we successfully demonstrated that our technique effectively mitigates matrix effects in hCOs‐derived samples through antibody‐based target specific detection (Figure S11 and Table S1, Supporting Information), further validation is required to confirm its robustness in clinically relevant environments. This study demonstrates that SERS‐based immunoassay is a powerful tool for exploring AD biomarker dynamics in hCOs, establishing a foundation for label‐free and non‐invasive detection techniques that maintain native biological activity. We believe that our biomarker detection strategy for hCOs using SERS will facilitate the robust clinical assessment of disease progression and offer direct insights into the *APOE*–tau axis in AD.

## 4 Experimental Section

### Fabrication of Gold‐Coated ZnO Nanorod Substrate

ZnO nanorods were fabricated by hydrothermal synthesis after the ZnO seed layer was deposited on a silicon (Si) wafer. The Si wafer was cleaned sequentially with acetone, isopropyl alcohol, and deionized water (DI water). Two seeding methods, evaporation and spin coating seeding, were evaluated to determine the optimal conditions for maximally enhancing the SERS signal. In the evaporation seeding method, 30 nm of ZnO was uniformly deposited on the cleaned wafer using a thermal evaporator at a rate of 0.1 nm s^−1^. For spin coating seeding, zinc acetate dihydrate (Zn(CH_3_COO)_2_ · 2H_2_O, 96 459, Sigma Aldrich) was dissolved into isopropanol solvent to make a 25 mm solution,^[^ ^76^ ^]^ followed by sonication for 30 min to prevent aggregation. The zinc acetate solution was spin‐coated on the cleaned Si wafer with 3000 rpm for 30 s and annealed on a hot plate at different temperatures (100, 200, and 300 °C) for 1 min, which was repeated 10 times. Then, the zinc acetate‐coated Si substrate was then baked at 350 °C for 1 h to remove any remaining organic solvents.

40 mm of zinc nitrate hexahydrate (Zn(NO_3_)_2_ · 6H_2_O, 228 737, Sigma Aldrich) and 25 mm of hexamethylenetetramine (HMTA, 33 233, Sigma Aldrich) or 1.2 mL of 28.0–30.0% ammonium hydroxide (NH_4_OH, 221 228, Sigma Aldrich) were prepared in 40 mL of DI water as reactant and stabilizer, respectively, for the hydrothermal reaction growth of ZnO nanorods.^[^ ^77^, ^78^ ^]^ The zinc acetated‐coated Si substrate was immersed into this solution and suspended with the seeded face downward in a hydrothermal reactor to avoid the accumulation of ZnO crystals on the surface. The hydrothermal reaction was performed at 90 °C in an oven for different reaction times (2, 3 or 4 h). After the reaction, the substrate was cleaned with DI water and dried with nitrogen gas. Finally, a 100 nm gold layer was deposited on the synthesized ZnO nanorods using a thermal evaporator at a rate of 0.1 nm s^−1^.^[^ ^78^ ^]^ The structural information of the ZnO nanorods was acquired using a scanning electron microscope (SU3800, Hitachi High‐Tech Science Corporation).

### Optimization of SERS Substrate and Antibody Immobilization

The Raman signal intensities of substrates synthesized under various conditions was compared using rhodamine 6G (R6G, R4127, Sigma Aldrich) to optimize the hydrothermal reaction conditions. R6G was uniformly coated onto the substrate using a spin coater set at 2500 rpm for 5 s. Subsequently, the SERS signal was collected using Raman spectroscopy with 12 mW of 785 nm laser (NS 200, Nanoscope Systems) with a 4X objective lens (NA 0.13). Furthermore, a monoclonal primary tau antibody (MN1000, Thermo Fisher Scientific) was immobilized on the optimized substrate via SERS signal through gold–thiol interaction. TCEP (Bond‐Breaker TCEP Solution, 77720, Thermo Fisher Scientific) solution was used to cleave disulfide bonds of the antibody, resulting in half‐antibody fragments with thiol groups. The antibody and TCEP solution were diluted in phosphate buffer saline (PBS, P3813, Sigma Aldrich) to make concentrations of 0.08–0.2 mg mL^−1^ and 0.02–0.04 mm, respectively.

For immobilization, the diluted antibodies and TCEP solution were mixed in a 1:1 volume ratio at different concentrations and incubated for 1 h at room temperature (RT) to generate half antibodies. 4 µL of this solution were dropped on the Au‐coated ZnO nanorod substrates and incubated overnight at 4 °C. The substrate was then washed twice with DI water for 15 min and dried with nitrogen gas. Unless otherwise specified, all subsequent washing and drying procedures were performed under the same conditions. Then, the substrate was immersed in 1x Pierce clear milk blocking solution (37587, Thermo Fisher Scientific) and incubated for 4 h at RT, followed by washing and drying. To ensure that the primary antibody was attached to the substrate, 4 µL of Alexa 488‐conjugated secondary antibody (A11001, Thermo Fisher Scientific) was dropped on the substrate and incubated it for 1 h at 4 °C. After washing and drying, the fluorescence intensity of the substrate was measured using a fluorescent microscope (Olympus) with a 10X objective lens (NA 0.3) to check the uniformity and attachment of the antibody on the substrate. Consequently, based on the SERS signal measurements, 0.16 mg mL^−1^ and 0.02 mm were adopted as the optimized concentrations for the monoclonal tau antibody and TCEP solution, respectively.

### Electromagnetic Simulation

To optimize the 3D prism‐like ZnO nanorod structures, Image‐J software was used to select four representative parameters from the SEM image of the ZnO nanorods substrate (i.e., diameter, XY angle, XZ angle, and height). Based on the SEM image, it was estimated that there were 64 nanorods in a 0.5 µm × 0.5 µm XY range. The X and Y coordinates for the nanorods positions, and each representative parameters were randomly sampled and applied to the 3D CAD software to design the 3D prism‐like structures (Figure S7e,f, Supporting Information). To fabricate the gold‐coated ZnO nanorods structures using 3D CAD software, a sphere‐like structure was added on top of the nanorod structures. Each structure, such as a ZnO nanorod‐like structure and a gold‐coated ZnO nanorod‐like structure, was imported into the FDTD electromagnetic simulation software for an accurate real‐condition simulation. To avoid structure overlapping, which induces inappropriate simulation results, each structure was imported in order and given different mesh orders, specifically ZnO nanorods were given higher priority number. Then, GNPs were imported 14.5 nm distant from the gold‐coated ZnO nanorods to make hotspots between them. The overall simulation region was 0.4 µm × 0.4 µm × 2.1 µm in size and mesh override region 0.4 µm × 0.4 µm × 0.65 µm with a 1 nm mesh size. A total‐field scattered‐field (TFSF) light source was used with 785 nm wavelength. The material parameters for ZnO were obtained from the literature^[^ ^79^ ^]^ and those for gold were obtained from Johnson and Christy.^[^ ^80^ ^]^

### Detection of Tau Protein via SERS

A monoclonal tau antibody‐conjugated substrate was prepared to detect the SERS signal from tau proteins following the procedure described in the previous section (“Section: Optimization of SERS substrate and antibody immobilization”). Purified fibrillar (TAU‐H5117, Acro Biosystems) and monomeric (TAU‐H5115, Acro Biosystems) tau proteins were diluted in PBS to various concentrations for calibration. Then, 4 µL of the diluted tau solution was applied to the substrate and incubated for 1 h at 4 °C, followed by washing and drying three times. The Raman signal was measured for each concentration to determine the LOD and create a calibration curve. To enhance the SERS signal, 20 nm gold nanoparticles (GNP, 741965, Sigma Aldrich) was conjugated with tau monoclonal antibody (sc‐32274, Santa Cruz Biotechnology) using gold–thiol interaction, creating hot spots between the substrate and the GNPs. The diluted antibody (0.2 mg mL^−1^) in PBS and 0.02 mm of TCEP were mixed in a 1:1 volume ratio and incubated for 30 min at RT to generate the thiol groups. Next, 20 µL of the 20 nm GNP was added to the TCEP‐treated antibody solution and incubated for 4 h at RT. After incubation, the solution was centrifuged at 12,500 rpm for 20 min, and the supernatant was removed to obtain the GNP‐conjugated antibody pellets. The pellets were resuspended in PBS, and 20 µL of the solution was incubated on the substrate for 1 h at 4 °C.^[^ ^39^ ^]^ After washing and drying, the tau protein signals were obtained using SERS.

The same protocol was used to detect tau proteins secreted by hCOs, using conditioned culture media. Specifically, SERS‐based detection was performed non‐invasively using only the collected media, without disrupting the organoids. Conditioned media were collected after 4 days of culture to ensure consistent measurements, as media replacement was performed at regular four‐day intervals. This media‐based detection approach enabled prolonged monitoring of tau secretion from the intact organoids and eliminated the need to expose the organoids to laser irradiation. Consequently, the measurement process did not affect the organoids themselves, as SERS was conducted on the collected media using a low‐intensity continuous laser (12 mW @785 nm) with a short exposure time of 1.5 s. All SERS measurements of tau proteins were performed after drying to maximize signal intensity at the substrate surface and to benefit from the pre‐concentration effect.^[^ ^81^, ^82^ ^]^ The only difference between tau protein detection from hCOs culture media was that the cultured dish for the day 100 hCOs was 2.5 times larger than that of day 40 hCOs, indicating that the concentration of tau protein in the day 40 hCOs culture medium was 2.5 times higher. Therefore, the day 40 hCOs culture medium was diluted 2.5‐fold with the culture media.

### Generation of hCOs from iPSCs and Collection of the Media

The previously published protocol was followed for generating iCOs.^[^ ^14^ ^]^ The iPSCs (*APOE2/E2*, BIONi010‐C‐6; *E3/E3*, BIONi010‐C‐2; *E3/E4*, BIONi010‐C; *E4/E4*, BIONi010‐C‐4; the European Bank for Induced Pluripotent Stem Cells) were maintained with mTeSR (ST100‐0276, STEMCELL Technologies), detached using ReLeSR (ST05872, STEMCELL Technologies), dissociated into a single cell, and collected at a concentration of 1500K cells. The collected cells were transferred to an AggreWell plate for EBs formation. EBs were collected on day 7 and seeded on the 96 well plate. Then, organoids were cultured in Neurobasal medium, GlutaMAX, B‐27 supplement minus Vitamin A, Penicillin‐Streptomycin (P/S), and basal Matrigel. Culture media of hCOs were collected on day 40 and 100, respectively, and stored at 70 °C. For using iPSCs and the generation of brain organoids, this project was approved by the Institutional Review Board of the Sungkyunkwan University (SKKU202407070‐UE001).

### Immunohistochemistry

The hCOs were washed with Dulbecco's phosphate buffer saline (DPBS), fixed with 4% PFA at 4 °C overnight, dehydrated in 30% sucrose at 4 °C for 72 h and then frozen in FCS 22 frozen section media (3 801 480, Leica) in the cryomold. The hCOs were frozen, sectioned, and permeabilized with 0.3% Triton X‐100 (X100, Merck) for 30 min at RT. Then, they were blocked with 5% horse serum (H0146, Sigma Aldrich) in 0.3% Triton X‐100 for 1 h at RT. Primary antibodies raised against MAP2 (1099‐MAP2, PhophoSolutions), SOX2 (RA 25 021, Neuromics), Thr181 (MN1050, Invitrogen), GFAP (PA1‐10004, Thermo), and DAPI (D9542, Sigma Aldrich) in blocking solution at a 1:500 ratio were applied at 4 °C overnight. A fluorophore‐conjugated secondary antibody diluted with bovine serum albumin (BSA) in DPBS at a 1:1000 ratio was applied for 1 h at RT. DAPI was diluted in DPBS at a ratio of 1:5000 and treated for 10 min and mounted. Microscopic images were obtained using a Leica Thunder Imager and analyzed with IMARIS software.

### RNA Sequencing Analyses for hCOs and Human Postmortem Brain Tissues

For hCOs, RNA sequencing data from the publicly available NCBI database (https://www.ncbi.nlm.nih.gov/bioproject/678865) with the accession number PRJNA678865 were used. For human postmortem brain tissues, the publicly available NCBI database (https://www.ncbi.nlm.nih.gov/bioproject/603192) with the accession number PRJNA603192 was used. For PRJNA678865, RNA sequencing analysis was performed according to our previous paper.^[^ ^14^ ^]^ For PRJNA603192, GEO2R analyzer (Accession number: GSE144254) was used to obtain differentially expressed genes (DEGs). To reveal *APOE*–tau axis genes, GO analysis was performed using ToppGene database (https://toppgene.cchmc.org/), and PPI analysis was performed using the STRING database (https://string‐db.org/).

## Conflict of Interest

The authors declare no conflict of interest.

## Author Contributions

Y.J., Y.K., and R.K. contributed equally to this work. I.K., J.P., and L.L. initiated and conceived the research; I.K., J.P., Y.J., and L.L. supervised, conceptualized, and identified authentic messages of the project; Y.J., Y.K., and R.K. designed the experiments; Y.K., R.K., S.L., D.N., and S.P. performed the experiments; Y.J., Y.K., R.K., D.L., J.H., and I.M. analyzed the data and drew figures; I.K., J.P., Y.J., Y.K., R.K., and L.L. wrote, edited, and reviewed the manuscript before submission.

## Code Availability

All relevant codes are available from the corresponding author upon reasonable request.

## Supporting information



Supporting Information

## Data Availability

The data that support the findings of this study are available from the corresponding author upon reasonable request.
