# Supplementary material for: Monitoring the Dynamics of Alzheimer's Disease Biomarkers and the APOE–Tau Axis via Human Cerebral Organoids with Immuno‐SERS
Source: Adv Sci (Weinh). 2025 May 30;12(35):e05660. doi: 10.1002/advs.202505660 (PMC12463057; doi:10.1002/advs.202505660)
Supplement: Supplementary file 1 — Supporting Information [file ADVS-12-e05660-s001.docx]

Supporting Information

**Monitoring the dynamics of Alzheimer's disease biomarkers and the APOE-tau axis via human cerebral organoids with immuno-SERS**

Yongjae Jo^#^, Youngjun Kim ^#^, Rian Kang ^#^, Seho Lee, Dang Du Nguyen, Soomin Park, Dongjoon Lee, Jong Won Han, Inhee Mook-Jung, Luke P. Lee*, Jong-Chan Park*, Inki Kim*


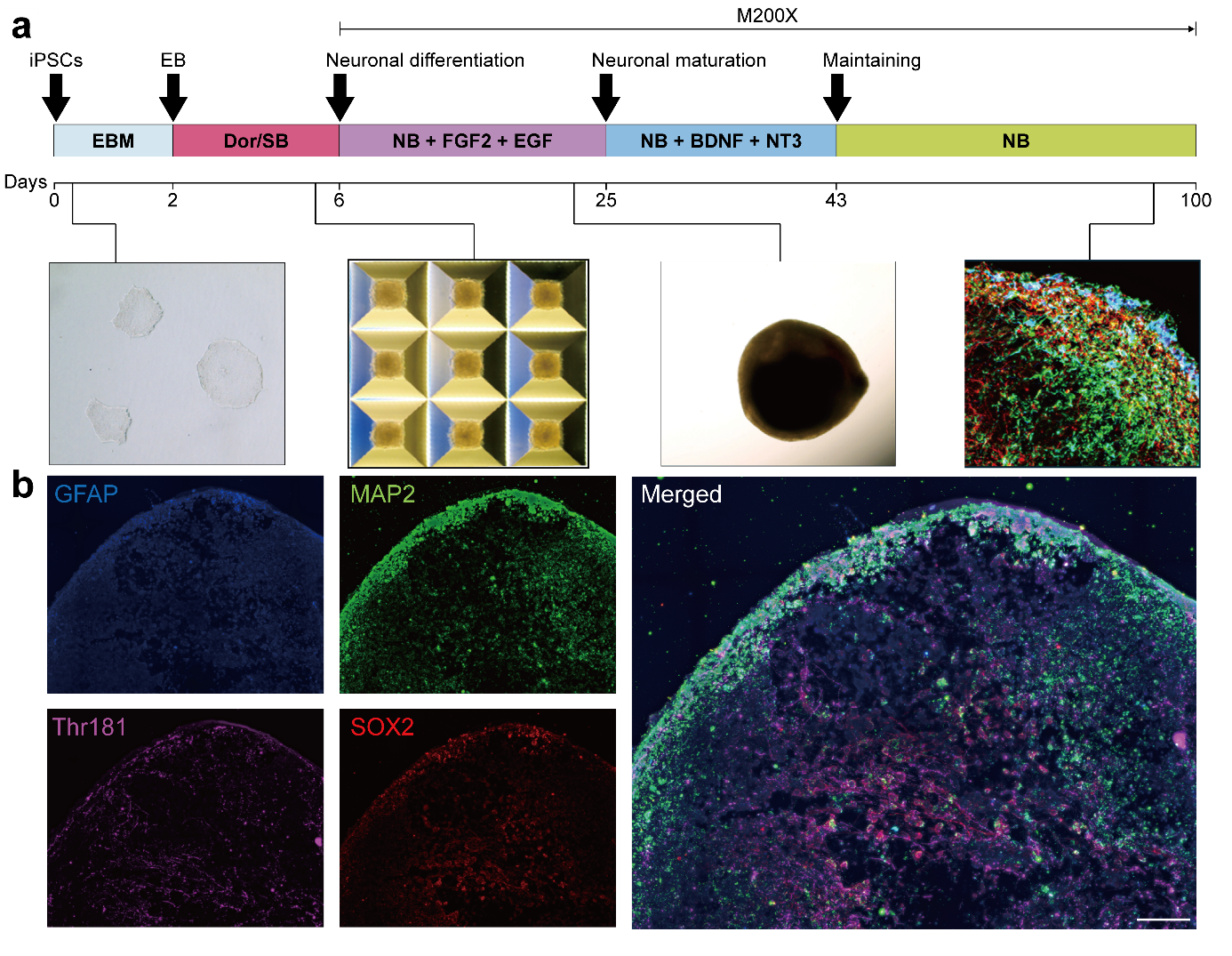


**Figure S1. Generation and validation of hCOs.** **a**, Schematic of hCOs generation with culture media collected at different days for analysis. **b**, IHC staining on day 83 hCOs using antibodies against GFAP, MAP2, Thr181, and SOX2. NB; neurobasal media, Dor; dorsomorphin, SB; SB431542, EGF; epidermal growth factor, FGF2; fibroblast growth factor 2 BDNF; brain-derived neurotrophic factor, NT3; neurotrophin-3, M; matrigel, EB; embryoid body, EBM; embryonic formation media. Scale bar: 100 μm (b).


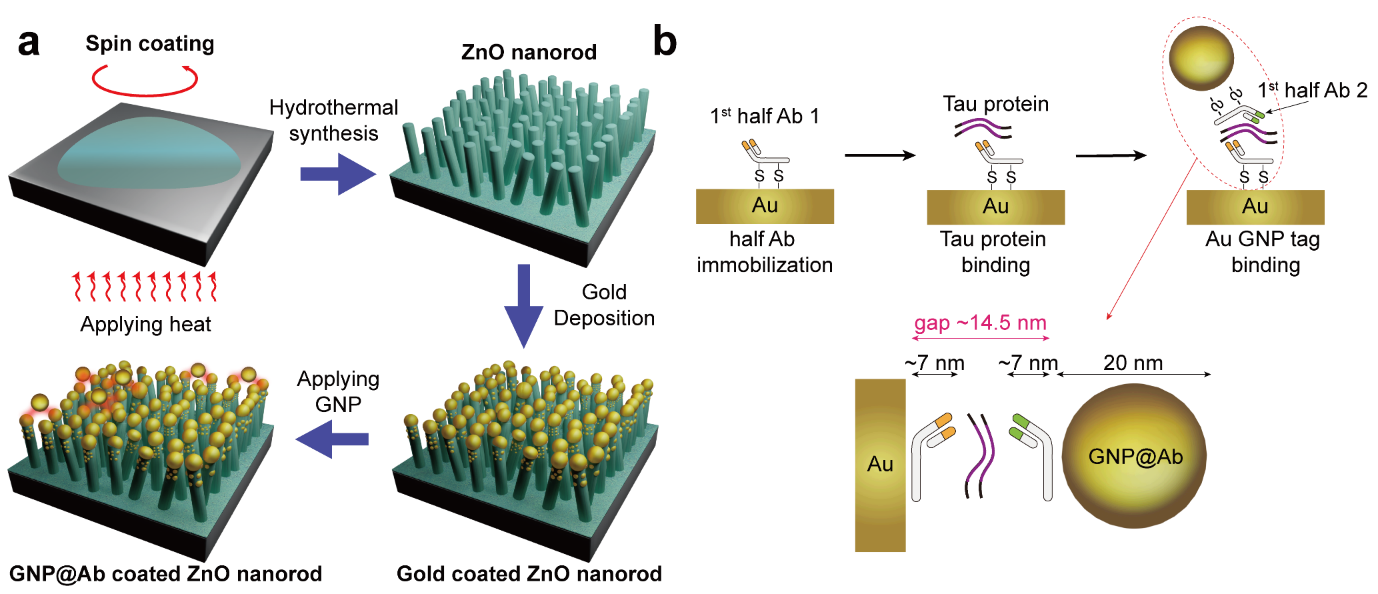


**Figure S2.** **Illustration of SERS based immunoassay fabrication. a,** Illustration of fabrication methods of GNP conjugated gold coated ZnO nanorods substrate. **b,** Illustration of half antibody fragments conjugation to SERS based immunoassay.


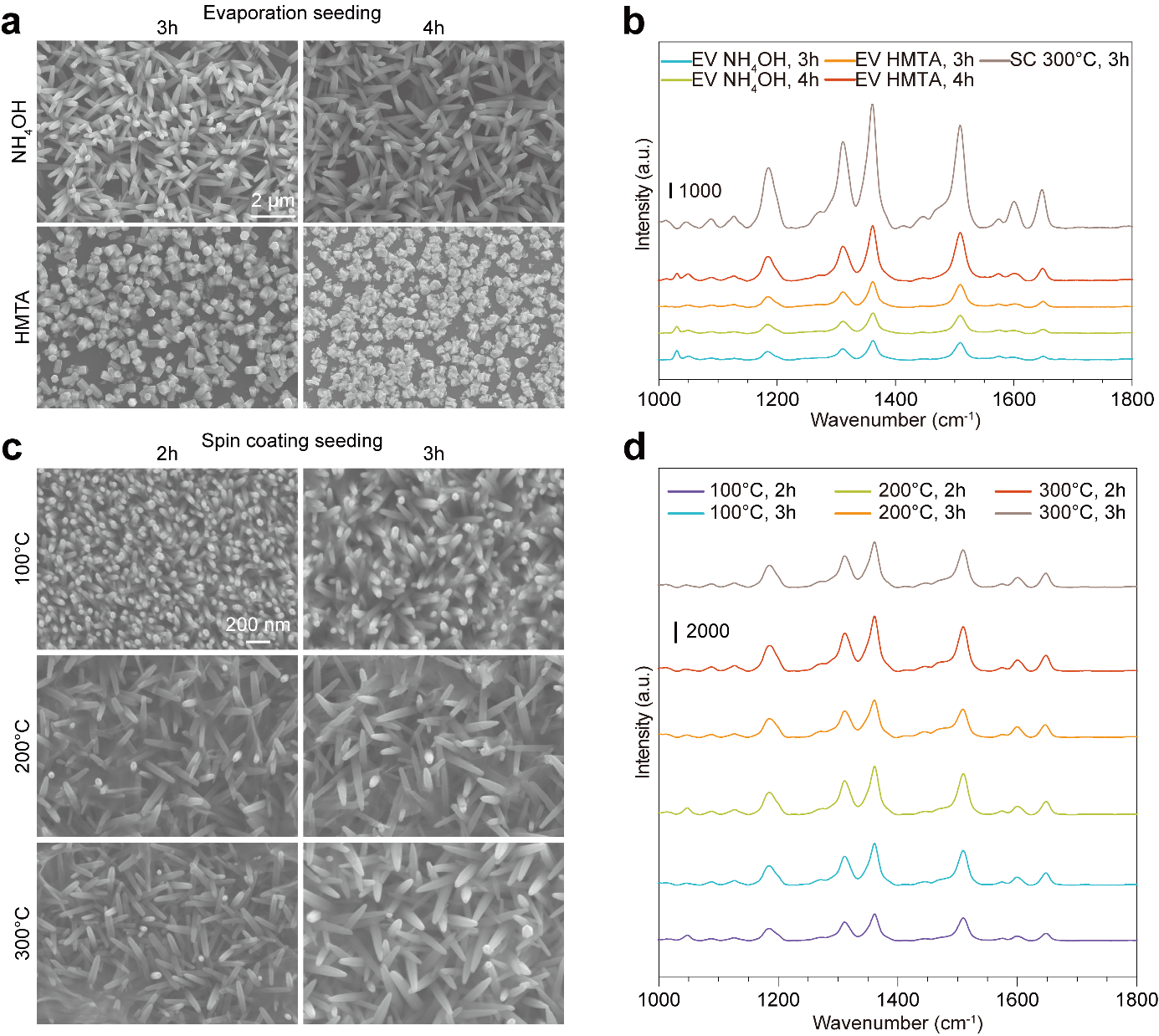


**Figure S3. Substrate Optimization. a,** SEM images of evaporation seeded ZnO nanorod with various conditions (3 h, 4 h: reaction time, NH_4_OH, HMTA: stabilizer). **b,** SERS spectrum of 1 μM R6G with different gold coated ZnO nanorods substrate. **c,** SEM images of spin coating seeded ZnO nanorod with various condition (2 h, 3 h: reaction time, 100 ˚C, 200 ˚C, 300 ˚C: annealing temperature) **d,** SERS spectrum of 1 μM R6G with different gold coated spin coating seeded ZnO nanorods substrate.


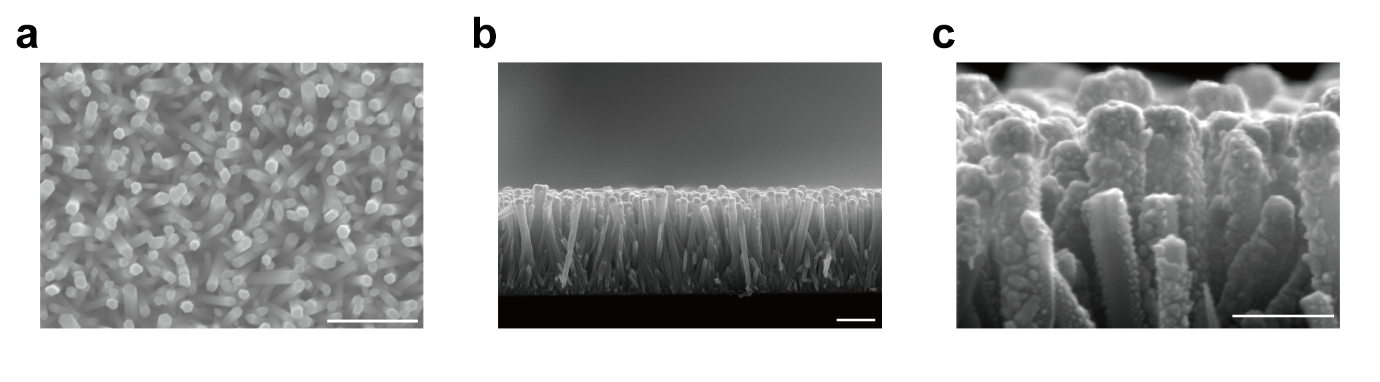


**Figure S4. SEM images of gold coated ZnO nanorods substrate. a,** Top view of 300 ˚C pre-heated and 3 hours reacted ZnO nanorods substrate with 100 nm gold deposition. Scale bar: 500 nm **b - c**, Vertical view of gold coated ZnO nanorods substrate. Scale bars: 500 nm (b) and 200 nm (c).


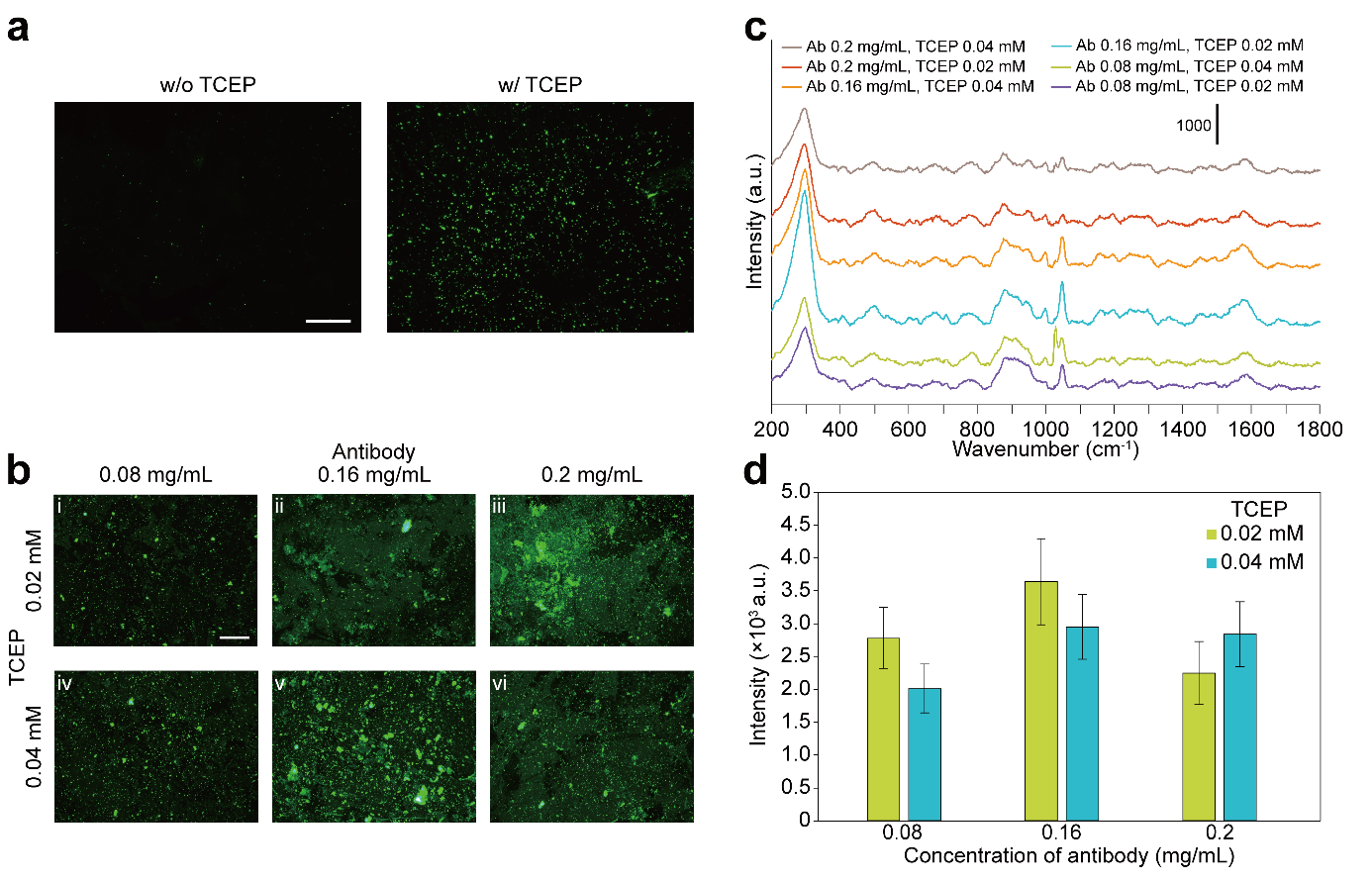


**Figure S5. Half antibody fragments Optimization. a,** Fluorescence microscopy of Alexa Flour 488 conjugated antibody with TCEP treatment. **b,** Fluorescence microscopy of Alexa Flour 488 conjugated TCEP treated half antibody fragments with different conditions. Scale bar: 200 μm (a, b). **c,** SERS spectrum of 1 μM Tau protein with different condition of half antibody fragments. **d,** Bar plots with standard deviation of SERS spectrum intensity at 300 cm^-1^. Data are presented as mean values ± s.d.


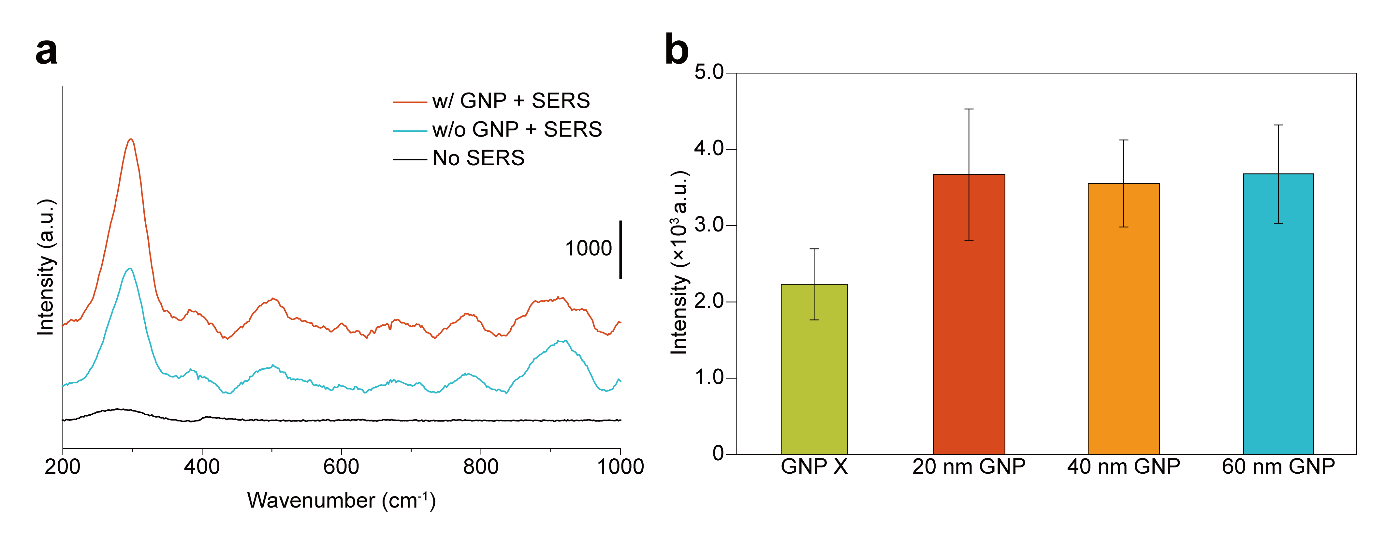


**Figure S6. SERS intensity comparison according to GNP applying. a**, SERS enhancement according to 20 nm GNP applying. **b**, SERS intensity comparison at 300 cm^-1^with different size of GNPs. Data are presented as mean values ± s.d.

**
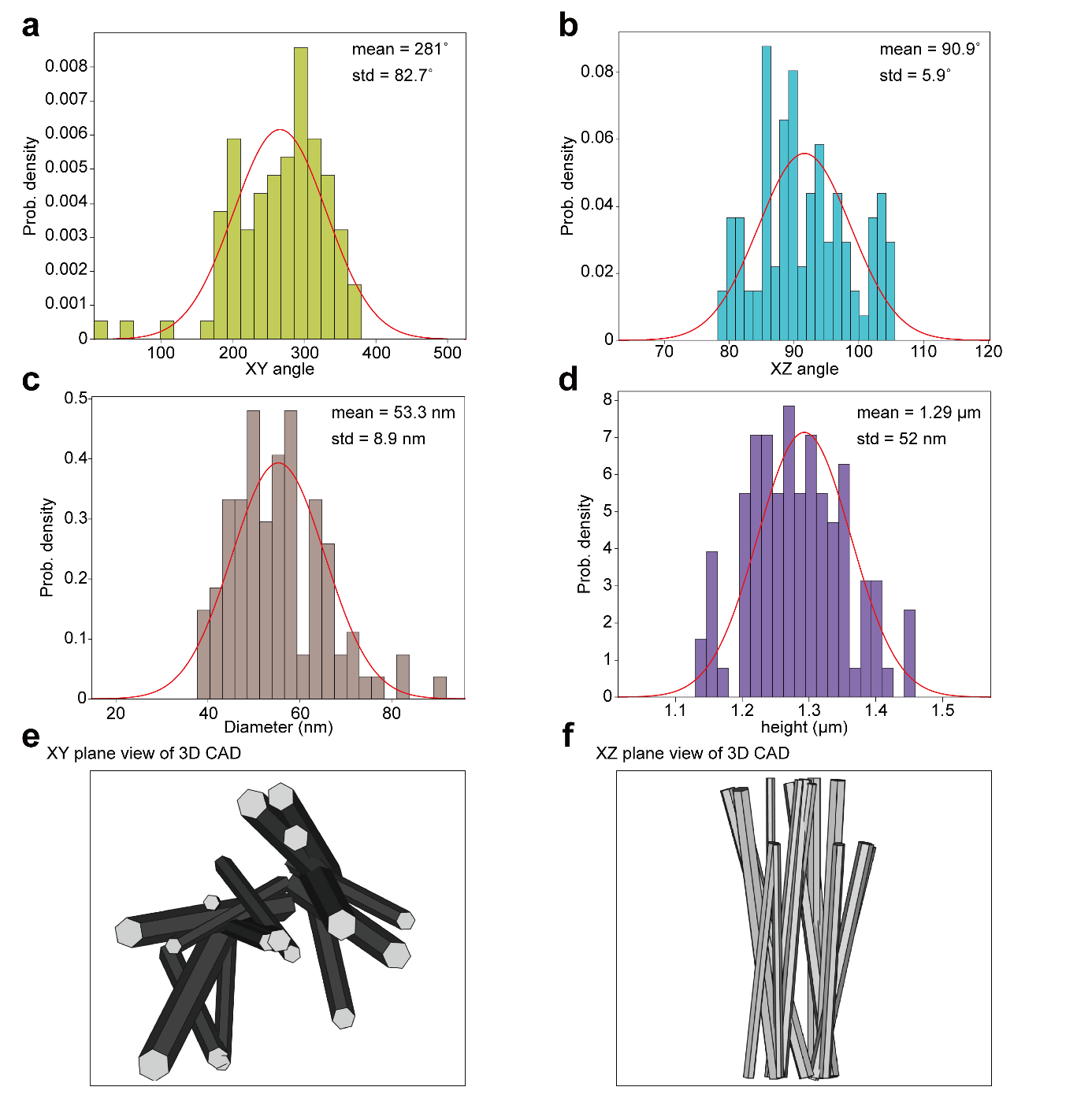
**

**Figure S7. Distribution of each parameter of ZnO nanorods and random sampled 3D hexagonal prism nanostructure with 3D CAD. a – d,** Normal distribution of each parameter, XY angle, XZ angle, Diameter, and Height, subsequently. **e – f,** 3D CAD structure with random sampled parameters from **Fig S7a – d**.


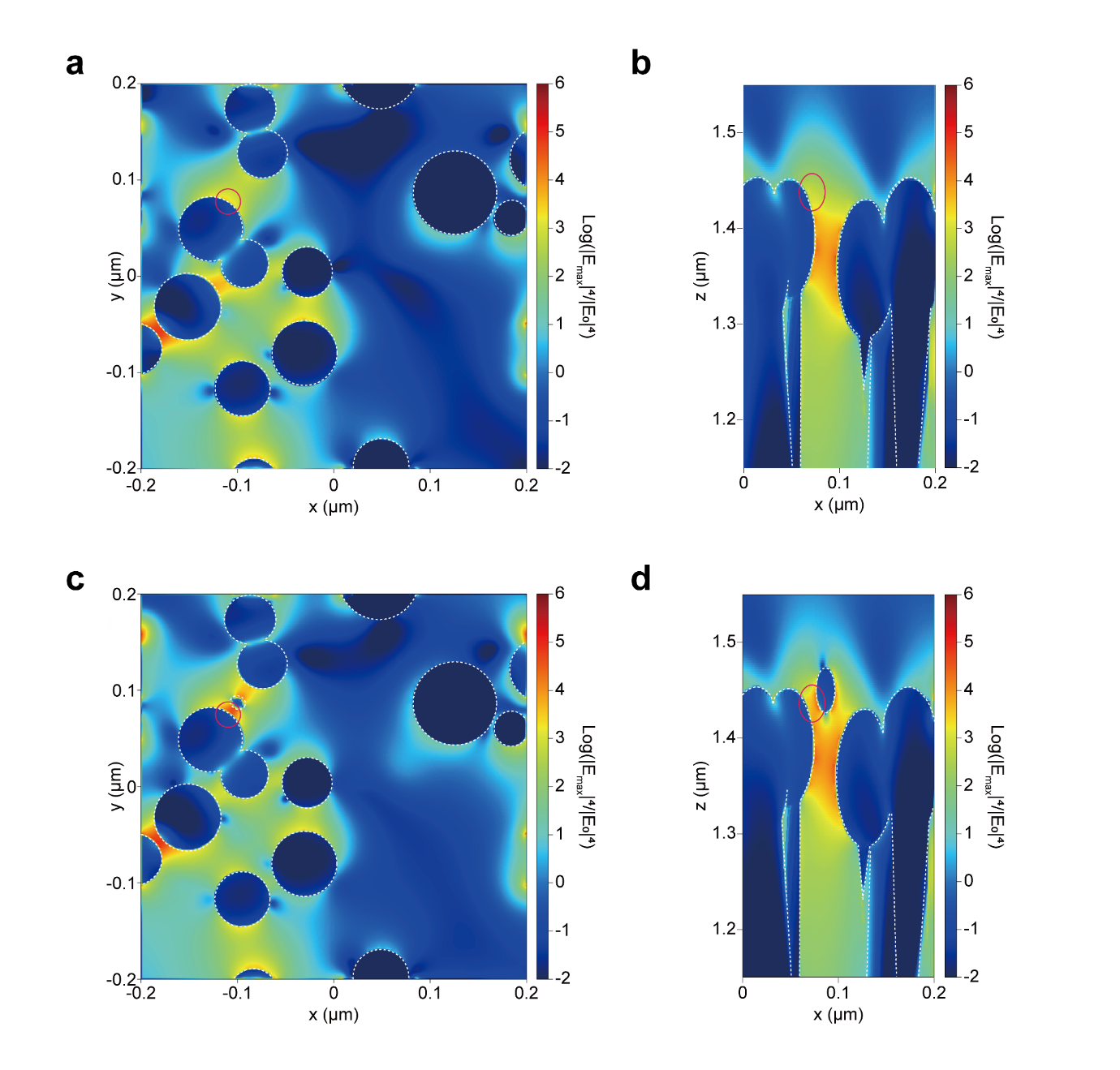


**Figure S8. FDTD simulation of SERS based immunoassay. a,** FDTD simulation for electric field enhancement without 20 nm GNP monitored in XY plane, showing 0.12 × 10^4^ enhancement factor. **b,** FDTD simulation for electric field enhancement without 20 nm GNP monitored in XZ plane, showing 0.14 × 10^3^ enhancement factor. **c,** FDTD simulation for electric field enhancement with 20 nm GNP monitored in XY plane, showing 0.76 × 10^5^ enhancement factor, 62.17 higher EF than without GNP. **d**, FDTD simulation for electric field enhancement with 20 nm GNP monitored in XZ plane, showing 0.12 × 10^5^ enhancement factor, 83.23 higher EF than without GNP.


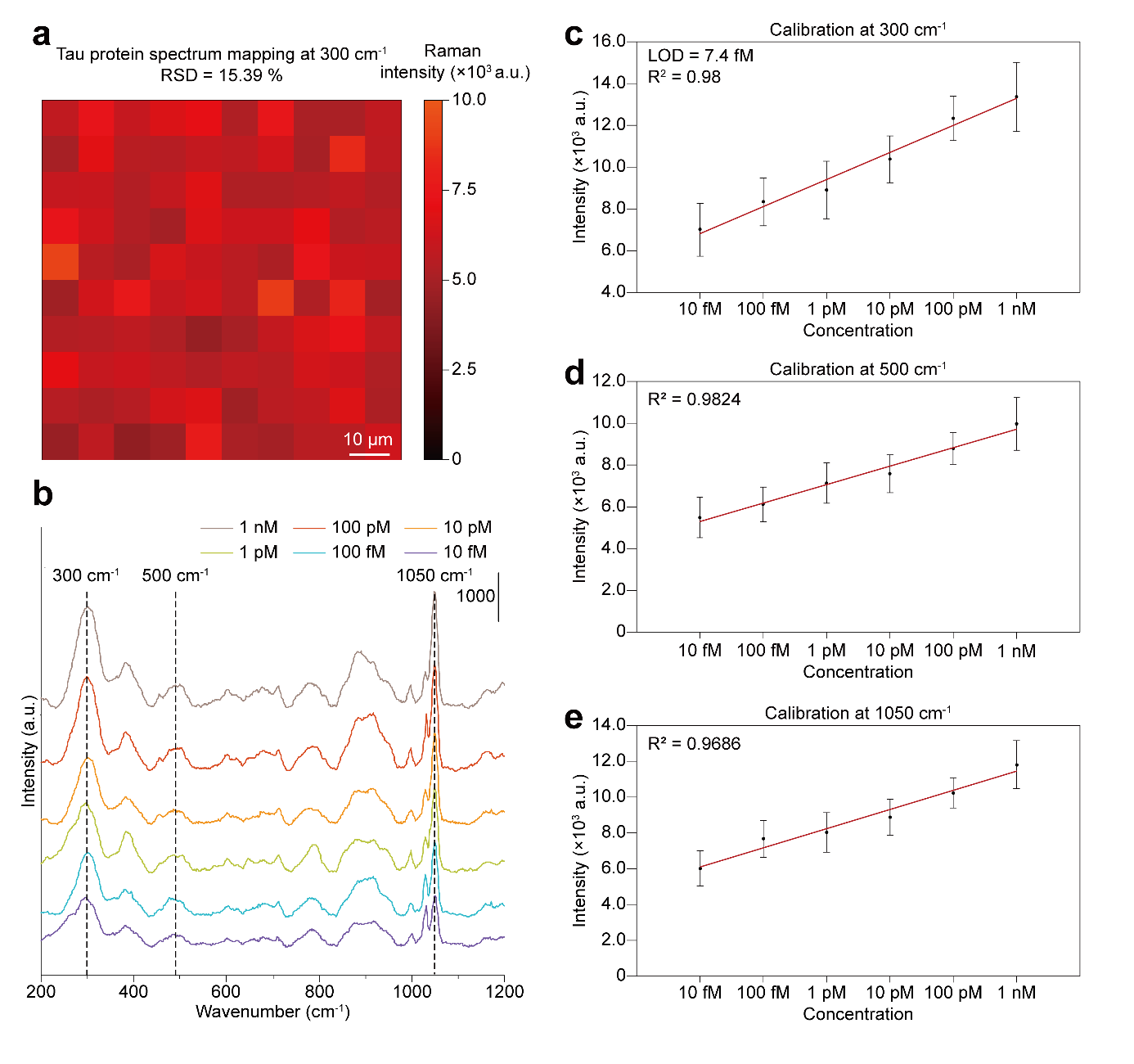


**Figure S9. SERS spectrum mapping and quantitative analysis of fibrillar tau protein. a,** SERS spectrum mapping with 1 mm × 1 mm, 100 spectra. RSD value is 15.39%, RSD: Relative Standard Deviation. **b,** SERS spectra from fibrillar tau protein from 10 fM to 1 nM concentration. **c,** Scatter plot and linear regression of SERS intensity for quantitative analysis from 10 fM to 1 nM concentration at 300 cm^-1^, corresponding to the Raman peak of gold-thiol bonding, which shows 7.4 fM limit of detection (LOD) with 0.98 R-squared value. Data are presented as mean values ± s.d. **d – e,** Scatter plot and linear regression of SERS intensity for quantitative analysis over the concentration range of 10 fM to 1 nM at 500 and 1050 cm^-1^, corresponding to the Raman peaks of disulfide bonding and phosphorylation, respectively.


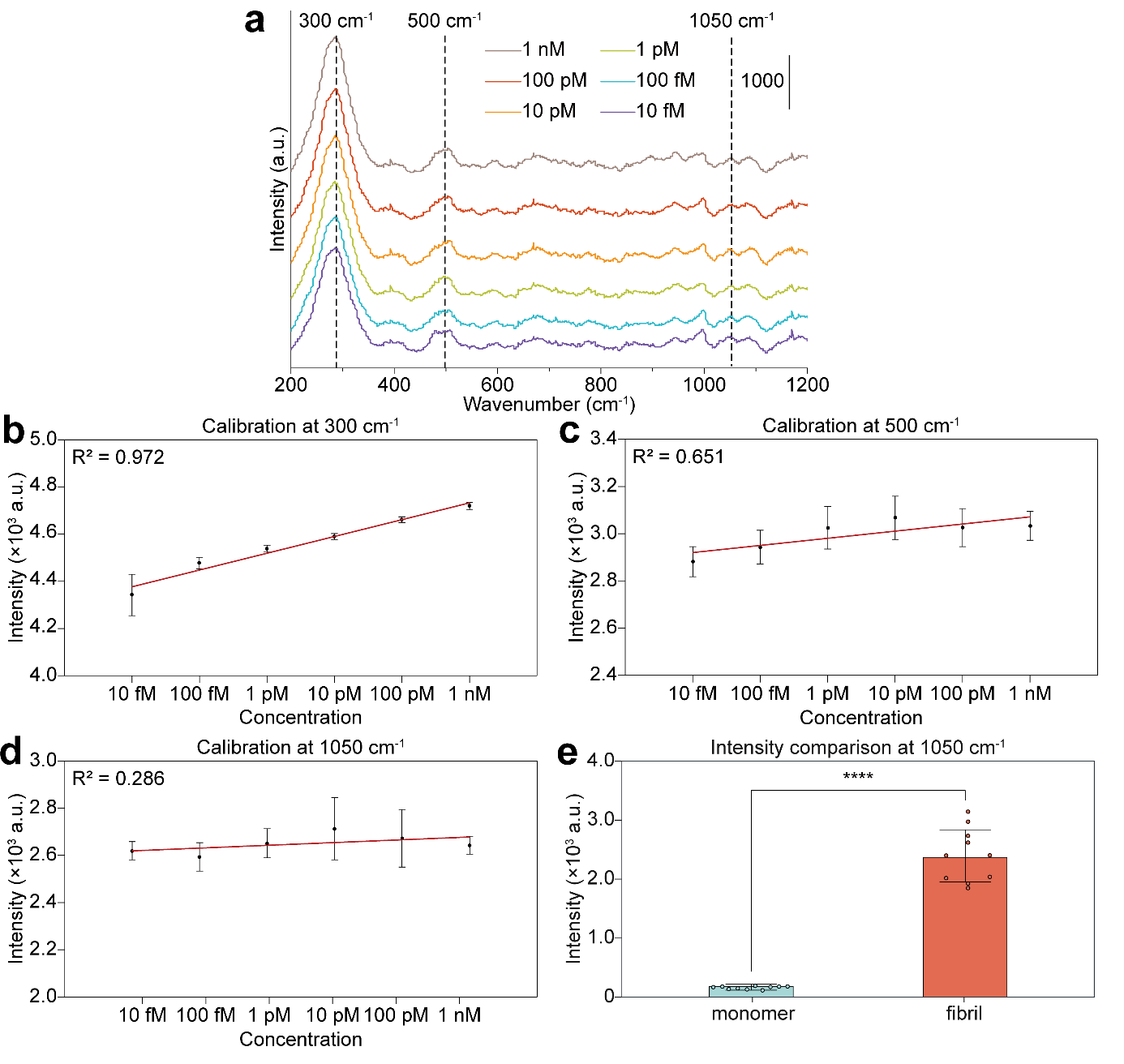


**Figure S10. Calibration of monomeric tau protein and SERS intensity comparison between fibrillar and monomeric tau protein. a,** SERS spectra from monomeric tau protein from 10 fM to 1 nM concentration **b - d,** Scatter plot and linear regression of SERS intensity for monomeric tau quantitative analysis over the concentration range of 10 fM to 1 nM at 300, 500 and 1050 cm^-1^, corresponding to the Raman peaks of total, disulfide bonding and phosphorylation, respectively. **e,** Comparison of SERS intensity at 1050 cm⁻¹ between monomeric and fibrillar tau proteins, showing that the fibrillar form exhibits approximately 13-fold higher signal intensity. Data are presented as mean values ± s.d. A two-tailed, unpaired t-test with Welch's correction was used. *****p* < 0.0001


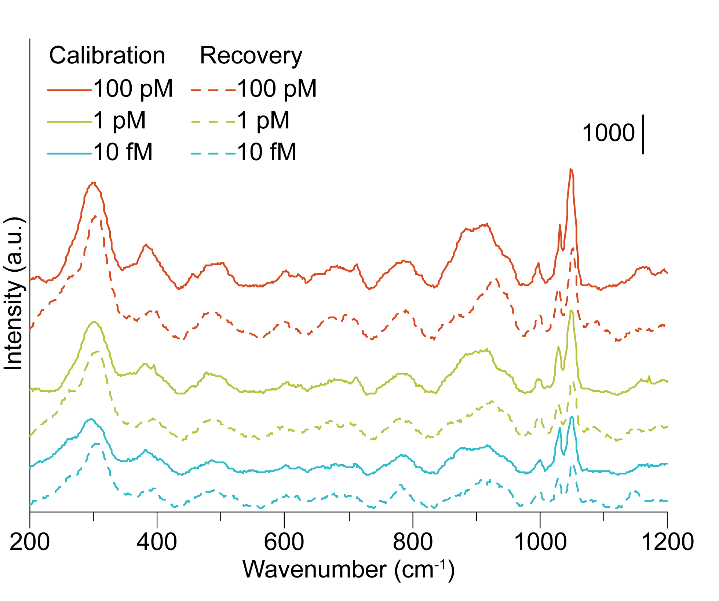


**Figure S11. Recovery test of tau detection.** Comparison of SERS spectra of tau protein dissolved in PBS (for calibration) and in organoid culture media (recovery test).


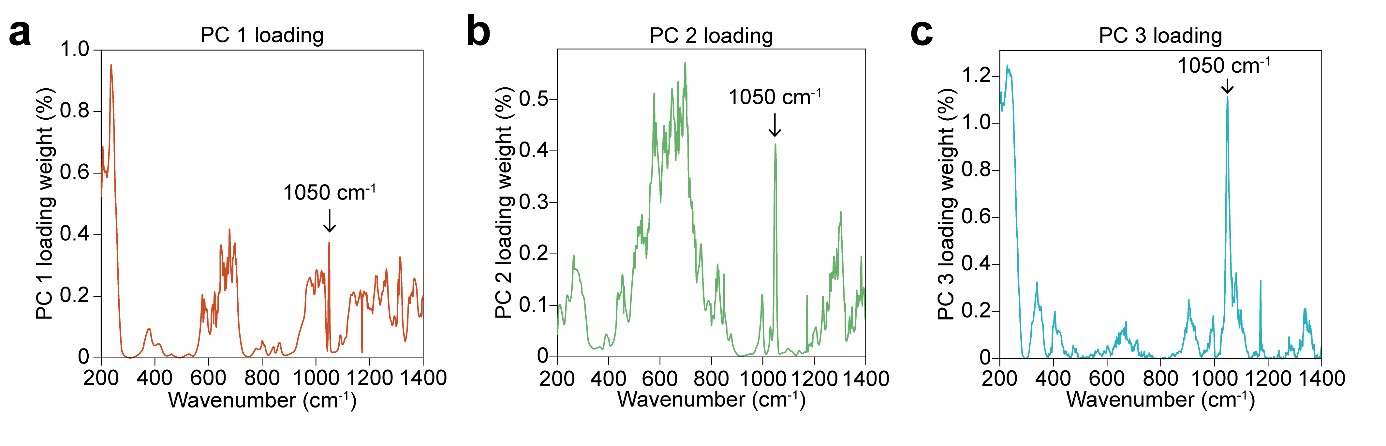


**Figure S12. Principal component loading plots for the discrimination of hCOs development stages. a – c,** Loading weights of the top three principal components, highlighting the key spectral features that contribute to distinguish the developmental stages of hCOs.


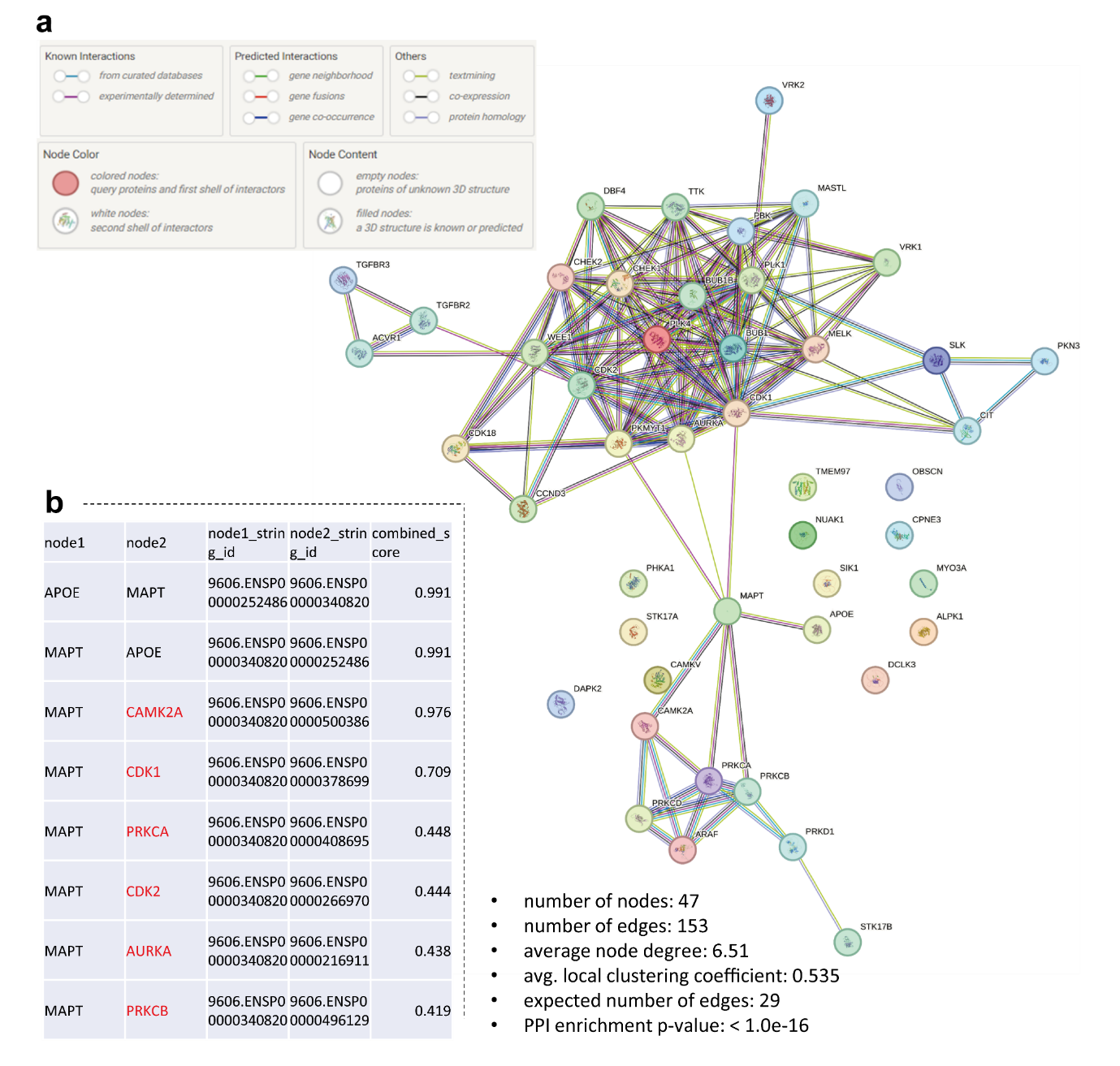


**Figure S13. Full protein-protein interaction (PPI) mapping result for *APOE3/E3* brain organoid and *E4/E4* brain organoid (related to Fig. 4c). a,** The differentially expressed genes (DEGs) between *APOE3/E3* brain organoid and *E4/E4* brain organoid with *MAPT* and *APOE* were used for PPI mapping via STRING database. **b** Interacting nodes (annotated genes) for APOE–tau axis. The red colored genes (*n* = 6) are possible APOE–tau axis genes.


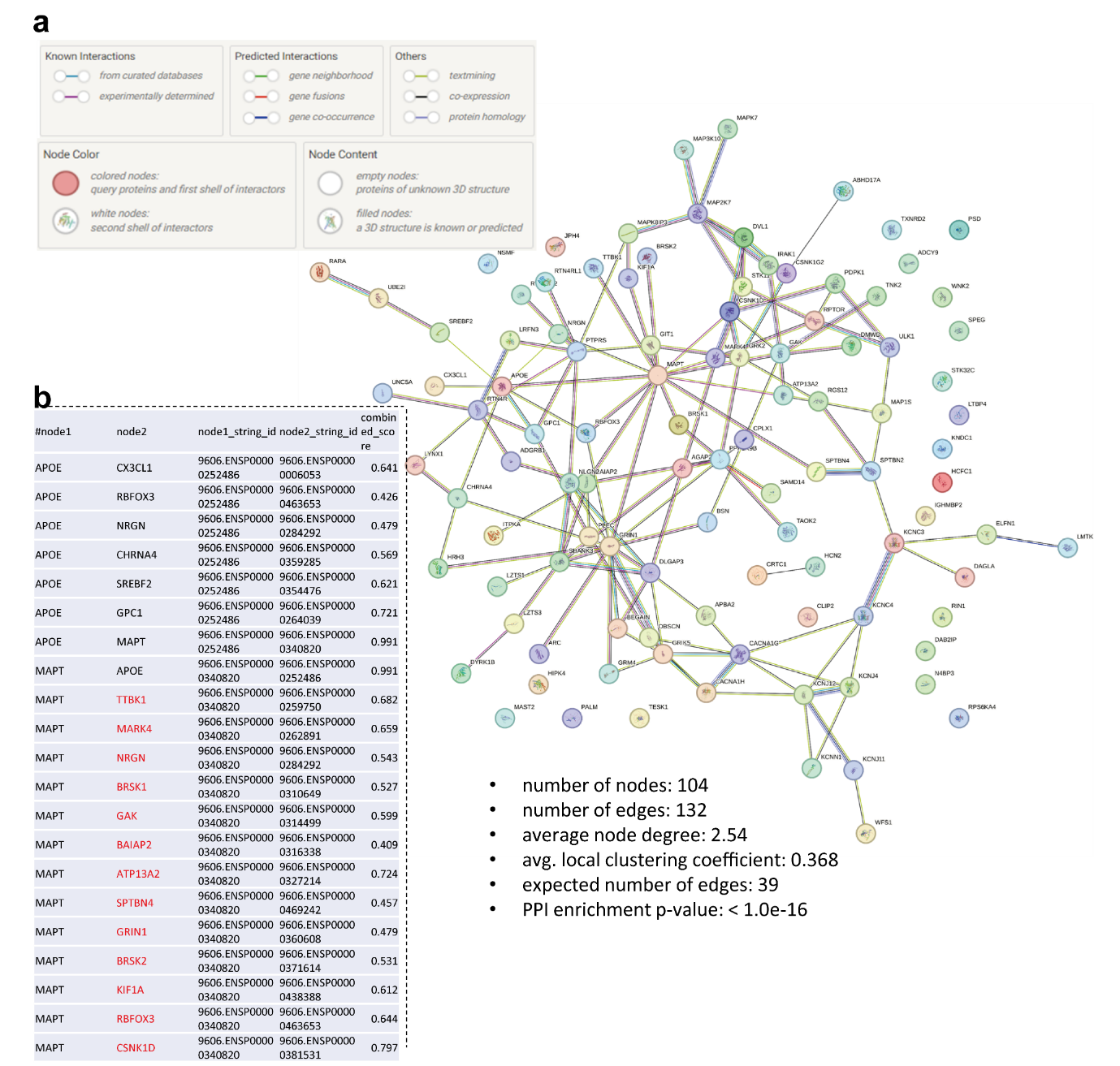


**Figure S14. Full protein-protein interaction (PPI) mapping result for human brain transcriptomic data (*APOE4* non-carriers vs *E4* carriers) (related to Fig. 4d). a,** The differentially expressed genes (DEGs) between *APOE4* non-carriers vs *E4* carriers with *MAPT* and *APOE* were used for PPI mapping via STRING database. **b,** Interacting nodes (annotated genes) for APOE–tau axis. The red colored genes (*n* = 13) are possible APOE–tau axis genes.


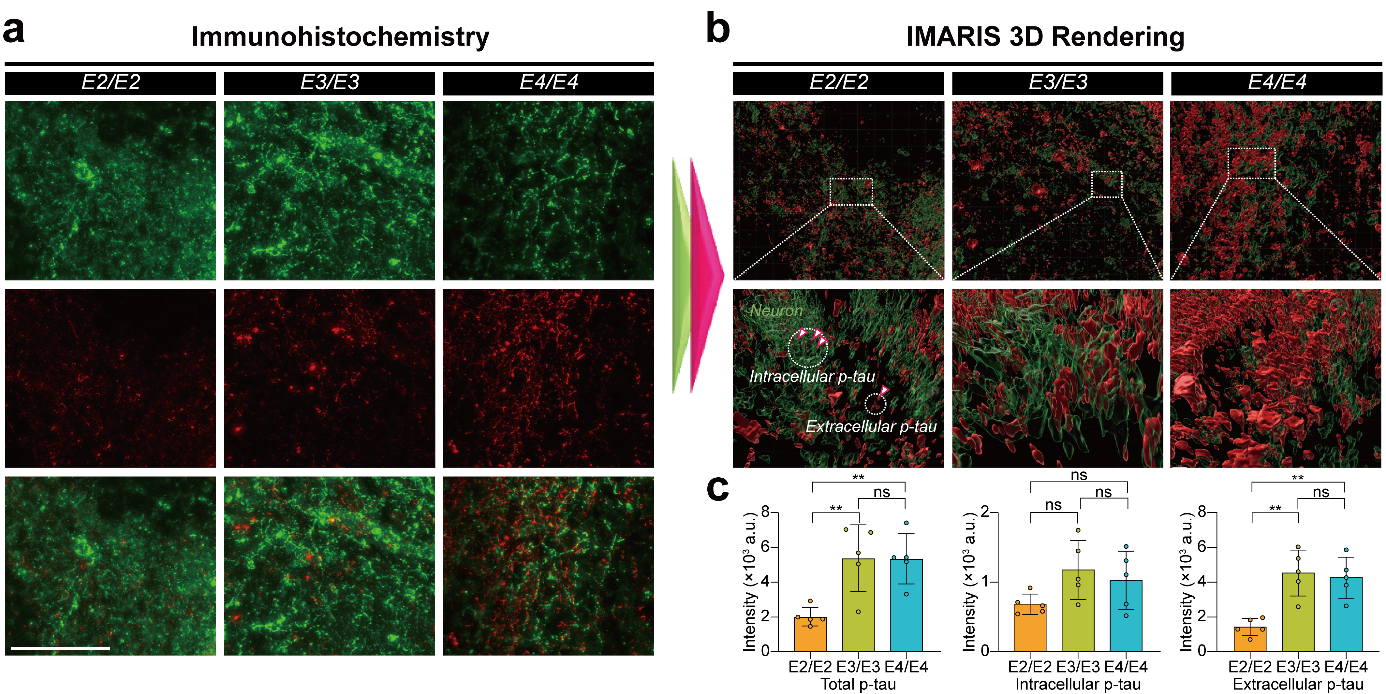


**Figure S15. hCOs IMARIS 3D rendered Immunohistochemistry a**, Immunohistochemistry showing neurons (MAP2) and p-tau (Thr181) in *APOE2/E2*, *E3/E3* and *E4/E4* hCOs. Green, neurons, MAP2; Pink, p-tau, Thr181. Scale bar: 100 μm. **b,** IMARIS 3D rendering images showing neuronal structure and p-tau. Green, neurons, MAP2; Red, p-tau, Thr181. **c,** Fluorescence quantification of total, intracellular, and extracellular p-tau. ANOVA with the correction of Tukey’s post hoc test for multiple comparisons. . ***p* < 0.01; ns, non-significance. Data expressed are as mean ± s.e.m.; *n* = 5, 5, 5 independent hCOs for *APOE2/E2*, *E3/E3*, *E4/E4* hCOs lines, respectively.

| **Table. S1** **SERS recovery test of fibrillar tau protein in hCOs culture media** | | | | |
| --- | --- | --- | --- | --- |
| Spiked  Concentration | Raman peak (cm^-1^) | Measured  Concentration  (SERS) | Recovery (%) | RSD (*n* = 5) (%) |
| 100 pM | 300 | 99.8 pM | 99.8 | 2.4 |
|  | 500 | 108.9 pM | 108.9 | 2.3 |
|  | 1050 | 96.8 pM | 96.8 | 3.1 |
| 1 pM | 300 | 1.00 pM | 100.3 | 1.2 |
|  | 500 | 0.97 pM | 97.4 | 1.5 |
|  | 1050 | 0.88 pM | 87.8 | 1.7 |
| 10 fM | 300 | 9.7 fM | 97.1 | 1.5 |
|  | 500 | 9.6 fM | 95.6 | 1.6 |
|  | 1050 | 8.7 fM | 87.0 | 4.4 |
